# Metal-Induced Energy Transfer (MIET) Imaging of Cell Surface Engineering with Multivalent DNA Nanobrushes

**DOI:** 10.1021/acsnano.3c10162

**Published:** 2024-01-17

**Authors:** Dong-Xia Wang, Bo Liu, Gui-Mei Han, Qingnan Li, De-Ming Kong, Jörg Enderlein, Tao Chen

**Affiliations:** † State Key Laboratory of Medicinal Chemical Biology, Tianjin Key Laboratory of Biosensing and Molecular Recognition, Frontiers Science Center for Cell Responses, Research Centre for Analytical Sciences, College of Chemistry, Nankai University, Tianjin 300071, P. R. China; ‡ III. Institute of Physics - Biophysics, Georg August University, 37077 Göttingen, Germany; § Cluster of Excellence “Multiscale Bioimaging: from Molecular Machines to Networks of Excitable Cells” (MBExC), Universitätsmedizin Göttingen, Robert-Koch-Strasse 40, Göttingen 37075, Germany

**Keywords:** metal-induced energy transfer, DNA nanostructure, membrane−membrane interaction, fluorescence lifetime, cellular adhesion, multicellular interactions

## Abstract

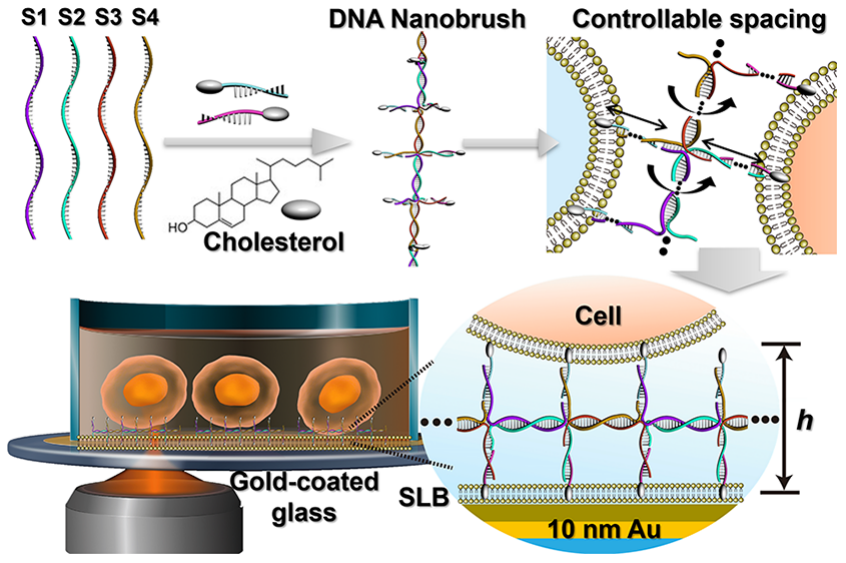

The spacing between cells has a significant impact on cell–cell interactions, which are critical to the fate and function of both individual cells and multicellular organisms. However, accurately measuring the distance between cell membranes and the variations between different membranes has proven to be a challenging task. In this study, we employ metal-induced energy transfer (MIET) imaging/spectroscopy to determine and track the intermembrane distance and variations with nanometer precision. We have developed a DNA-based molecular adhesive called the DNA nanobrush, which serves as a cellular adhesive for connecting the plasma membranes of different cells. By manipulating the number of base pairs within the DNA nanobrush, we can modify various aspects of membrane–membrane interactions such as adhesive directionality, distance, and forces. We demonstrate that such nanometer-level changes can be detected with MIET imaging/spectroscopy. Moreover, we successfully employed MIET to measure distance variations between a cellular plasma membrane and a model membrane. This experiment not only showcases the effectiveness of MIET as a powerful tool for accurately quantifying membrane–membrane interactions but also validates the potential of DNA nanobrushes as cellular adhesives. This innovative method holds significant implications for advancing the study of multicellular interactions.

Cell–cell interaction is a vital physiological process in multicellular organisms.^1^ Accurate control of cell–cell interaction presents a way to study and manipulate various cellular processes, thus benefiting the development of cell-based theranostics and tissue engineering.^2−4^ Cell surface engineering strategies hold great potential in regulating cell–cell interactions by modifying the surface with various functional materials, such as proteins, nucleic acids, nanoparticles, or polymers.^5−8^

While cell surface-engineered materials or surface-engineering strategies are experiencing thriving development,^9,10^ a crucial aspect that has been largely overlooked is the characterization of their ability to regulate the spacing between membranes. This oversight can be attributed to the limited availability of characterization tools specifically designed for this purpose. However, the intermembrane spacing^11−13^ and cell fusion^14,15^ are crucial for an understanding of cell membrane surface engineering processes. Therefore, it is imperative to develop suitable tools and techniques to investigate nanomaterial interactions at nanometer distances with cellular membranes, as this knowledge is essential for comprehending cell membrane surface engineering processes.

Various methods and techniques, such as cryogenic transmission electron microscopy (cryo-TEM),^16,17^ neutron reflectometry (NR),^18^ or super-resolution fluorescence microscopy^19^ have been employed to quantify the distance between different membranes. However, those methods and techniques are limited in their ability to observe dynamic changes during adhesion processes.^20^ Although reflection interference contrast microscopy (RICM) and total internal reflection fluorescence microscopy (TIRFM) have the potential to measure membrane dynamics,^4,21,22^ their application to nucleated cells is risky due to the presence of complex components inside the cell and proteins on the membrane. These factors can lead to ill-defined variations in the refractivity, making the measurements unreliable. Förster resonance energy transfer (FRET), wherein an emitter (the donor) transfers its excited state energy to nearby molecule(s) (the acceptor(s)), stands as a robust technique for discerning subnanometer distances. Recent advancements in FRET, leveraging quenchers such as a graphene oxide layer, blue dextran, and trypan blue, have facilitated the exploration of membrane dynamics and interactions between membranes and proteins.^23−25^ Nevertheless, it is crucial to note that owing to its inherently narrow working distance, typically smaller than 10 nm, FRET may not be the optimal choice for studying membrane–membrane interactions.

Recently, our group has developed a method called metal-induced energy transfer (MIET) to precisely determine the axial position of a fluorescent single molecule above a metal film.^26,27^ The principle of MIET is based on the energy transfer of the excited state energy of an excited fluorophore to surface plasmons in the metal film. This energy transfer is extremely distance-dependent and leads to a distance-dependent modulation of fluorescence lifetime and intensity.^28^ Due to the broad absorption spectra of metals, the energy transfer from a fluorescent molecule to the metal film takes place with high efficiency across the full visible spectrum. Thus, any dye in the visible spectral range will be affected by MIET, and its measured fluorescence lifetime can be converted to a distance of the emitter from the metal surface. MIET has been used for investigating various systems, from whole cells to organelles, and to determine the axial position of individual molecules with a precision of ca. 3 nm.^29,30^

Here, we use MIET imaging/spectroscopy to precisely measure the intermembrane distance in DNA-nanostructured modulated membrane systems with nanometer-scale accuracy (Scheme 1). To achieve this, we developed a DNA-based adhesive called a DNA nanobrush. One of the key advantages of this DNA adhesive is its versatility in design, which allows for the manipulation of valence states to modify intercellular forces between cell membranes. Additionally, it provides the ability to adjust the number of base pairs on the brush backbone and tentacles, thereby enabling controlled regulation of the adhesive directionality and distance. We demonstrate that MIET can effectively and accurately measure nanometer-sized changes in the distance between cell membranes decorated with DNA nanobrushes. Furthermore, we applied MIET to monitor the adhesion process between cellular membranes induced by the DNA nanobrush. Our results not only confirm the potential of MIET as a powerful tool for studying intercellular interactions but also highlight the potential of our DNA nanobrush as a molecular glue for cellular assembly.

**Scheme 1:**
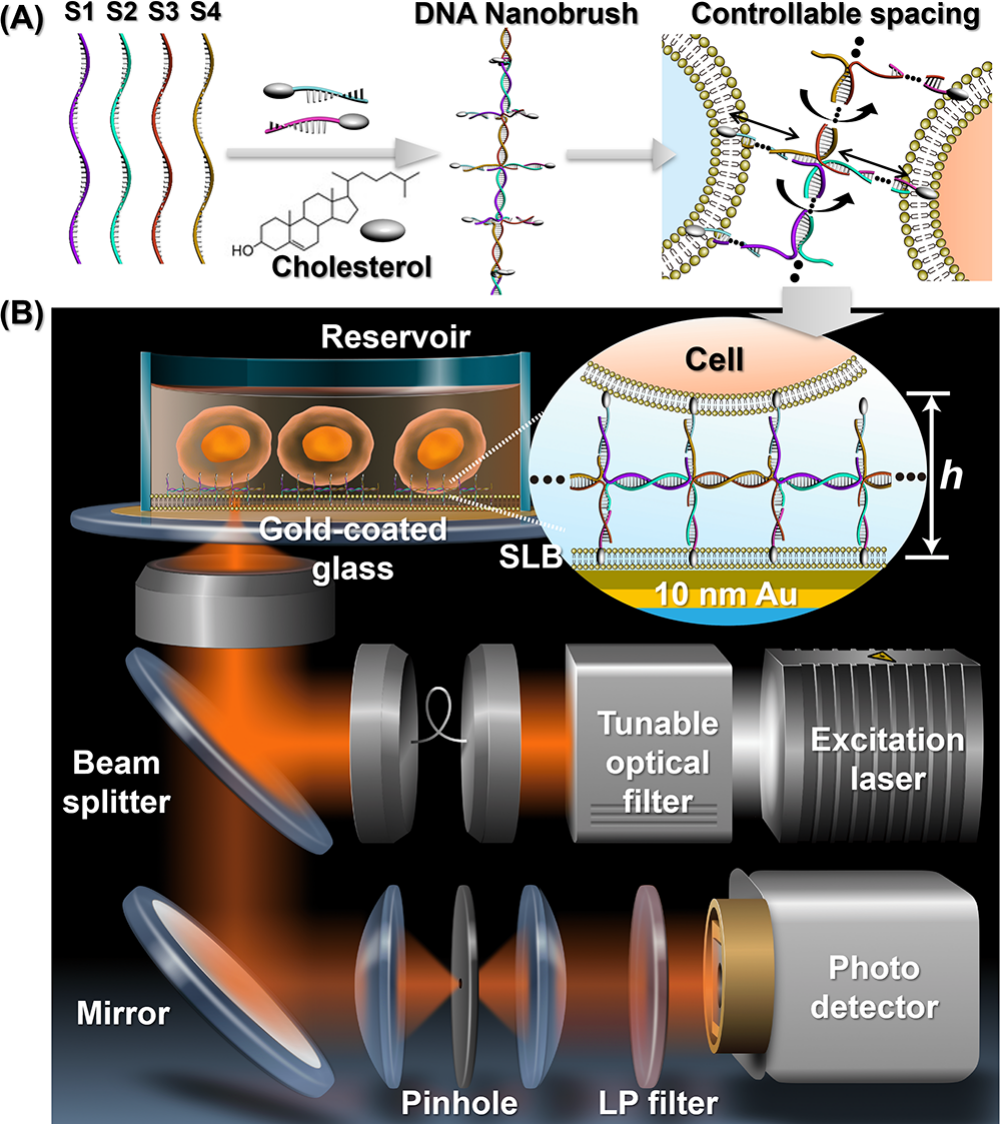
Multivalent DNA Nanobrush Engineered on a Cell Membrane Surface for Precisely Quantifying Intercellular Interactions via Metal-Induced Energy Transfer

## Results and Discussion

### Synthesis and Characterization of Multivalent DNA Nanobrushes

**Figure 1:**
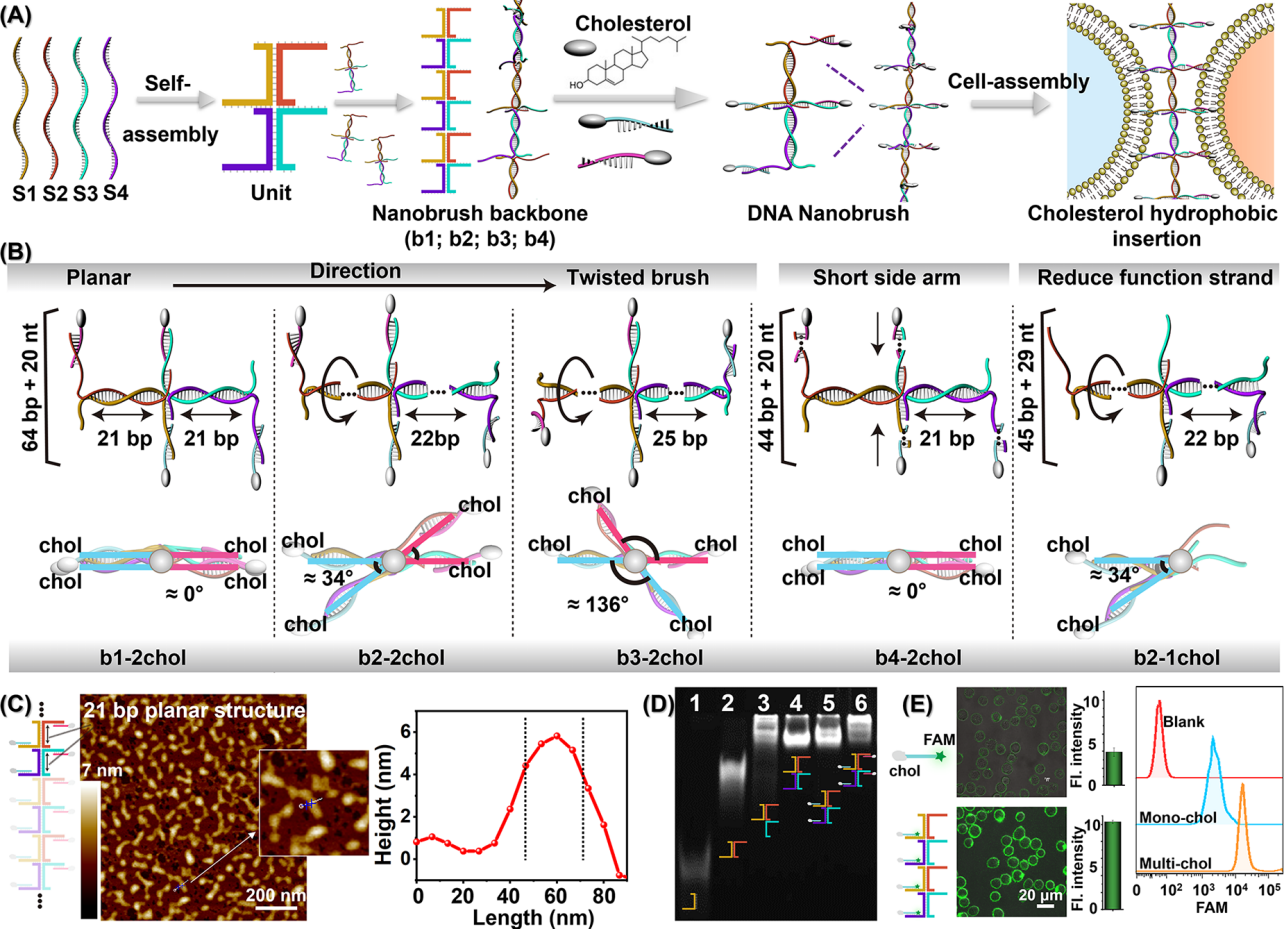
Synthesis and characterization of the DNA nanobrushes. (A) Schematic illustration of cell surface engineering with a DNA nanobrush. (B) Structural illustration of DNA nanobrushes (b1, b2, b3, b4) with different twist angles due to changes in backbone, side arms, and functional strands. (C) AFM images of a DNA nanobrush (b1-2chol). The right panel shows a linear cross-section of the height profile of a nanobrush, displaying dimensions of approximately 6 nm in height and 30 nm in diameter. (D) PAGE assay of different DNA samples. From lanes 1 to 6: S1; S1+S2; S1+S2+S3; S1+S2+S3+S4 (brush backbone, b1); b1-1chol; b1-2chol. (E) Membrane anchoring capacity of monovalent-cholesterol and multivalent-cholesterol DNA nanobrushes. The middle panels show fluorescence intensity images. The right panel shows a flow cytometry analysis of monovalent- and multivalent cholesterol. More than 100 cells per group were measured and then analyzed with ImageJ, and for each group, three independent measurements were performed.

A series of DNA nanobrushes were designed and synthesized for cell membrane surface engineering (see the Supporting Information Experimental Section and Table S1). The backbone units of these DNA nanobrushes were composed of four short single-stranded DNA strands (S1, S2, S3, and S4) linked in series to form a linear core (Figure 1A). The backbone units assembly principle is similar to Holliday junctions.^31,32^ The number of base pairs (bps) in the linear backbone is designed to be 21 bp (b1), 22 bp (b2), and 25 bp (b3), respectively. Theoretically, each base pair contributes about 0.34 nm of length and about 34.3° of twist to the growing helix,^33^ resulting in a helical twist of 10.4 base pairs/turn (bp/turn) for B-form DNA.^34^ Thus, the base pair numbers that one uses determines the arrangement and direction of the functional strands: gradually from a planar (21 bps, b1) to a twisted brush (b2 and b3). To shorten the distance of cell spacing, we also designed a short side arm nanobrush (b4) based on the b1 backbone (Figure 1B and Figure S1). From the nanobrush backbone, numerous side arms (tentacles) can extend that can hybridize with cholesterol-labeled complementary strands, thereby introducing cholesterol functional groups that can link to a membrane. Using different single-stranded sequences on the side arms, it is possible to artificially control the number and position of the introduced cholesterol groups. Here, we employ two different single-stranded sequences on the side arms, each capable of hybridizing with its cholesterol-labeled complementary strand. When hybridized with one of these complementary strands, cholesterol is added to half of the side arms (b1/2/3/4-1chol). When hybridized with both complementary strands simultaneously, cholesterol is added to all side arms (b1/2/3/4-2chol). Compared to other rigid DNA structures such as DNA origami or amphiphilic DNA probes, the DNA nanobrush offers more flexibility, which is advantageous for interaction with highly flexible membranes, even when they exhibit high curvature and dynamics.^35,36^

First, the structure of the DNA nanobrush was evaluated by atomic force microscopy (AFM). Using b1-2chol DNA nanobrush as an example, as shown in Figure 1C, b1-2chol shows a flexible linear structure with a length of ∼100 nm, demonstrating the successful assembly of the nanobrush. The height of the brush is only ∼6 nm, and the width is ∼30 nm, which suggests a planar structure for the b1 DNA nanobrush. Polyacrylamide gel electrophoresis (PAGE) demonstrates the successful self-assembly of a nanobrush by rapid programmable annealing (Figure 1D). By sequentially adding DNA from S1 to S4, the gel migration of the DNA mixture slows down gradually (lanes 1–4). After adding the functional side arm strands (lanes 5 and 6), all gel bands with delayed migration correspond to samples with successful assembly.^37,38^ Structural characterization results about other nanobrushes can be found in Figure S2. Next, we evaluated the anchoring ability of the nanobrush to the cell membranes. We attached FAM fluorophores to cholesterol-labeled DNA strands. CCRF-CEM, a T lymphoblastoid cell line, was used as a model cell line. With multivalent hydrophobic vertices, DNA nanobrushes (b1–1chol) showed a strong membrane-anchoring ability without becoming internalized. Over 90% of the probe was retained on the membrane even after incubation in 10% FBS-containing medium for 1 h. However, the monovalent-cholesterol strand (single-strand DNA labeled cholesterol) rapidly dissociated from the cell membrane after 30 min (Figure S3). As evaluated from the fluorescence intensity, multivalent-cholesterol DNA nanobrushes are almost 2.7 times larger in thickness than monovalent-cholesterol ones (Figure 1E). This was further confirmed with flow cytometry.^39^ These results demonstrate that multivalent cholesterol can stably anchor nanostructures on the membrane surface, providing a stable anchoring method for subsequent MIET measurements.

### Nanobrush for Cell Surface Engineering

**Figure 2:**
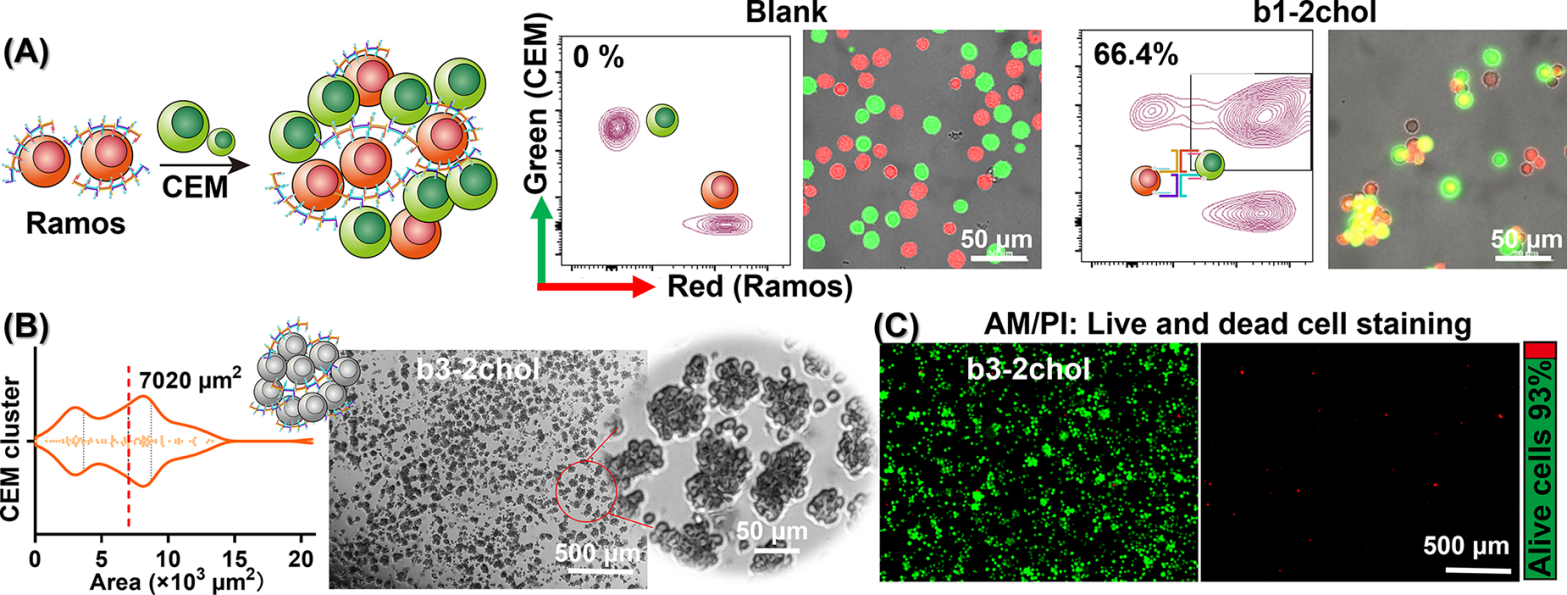
Nanobrush for cell surface engineering. (A) DNA nanobrush (b1-2chol) induced heterotypic cell aggregation. Assembly characterization was performed using confocal laser scanning microscopy (CLSM) and flow cytometry, respectively. Green: CCRF-CEM cells; Red: Ramos cells. (B) Aggregates of CCRF-CEM cells assembled with b3-2chol. The cross-sectional area distribution of homogeneous CEM cell spheroids after assembly for 24 h. The area of 100 cell clusters was measured using ImageJ. Characterization was performed using bright field cell microscopy. (C) Live–dead staining image of CEM cell spheroids after 36 h. Alive and dead cells were stained with Calcein-AM (green) and PI (red), respectively. The bar on the right shows the quantified optical density of the fluorescence images.

We utilized our nanobrush to guide and program cell–cell binding versatility using CCRF-CEM cells and Ramos cells (human B lymphoma cells) as test samples. We optimized bonding conditions using the planar structure (b1-2chol) with a nanobrush backbone of 21 bp (Figure S4). Control measurements attest that cell assembly did not occur under conditions of: (1) no addition of nanobrushes; (2) cholesterol molecules alone; (3) cholesterol-modified ssDNA alone; and 4) addition of DNA nanobrushes lacking the cholesterol group (Figure 2A and Figure S5). Moreover, the inclusion of monocholesterol modified DNA nanobrushes resulted in only a 36.8% assembly efficiency. However, in the presence of a multivalent cholesterol-modified DNA nanobrush, the assembly efficiency reached 66.4% (CEM:Ramos = 1:1), indicating that our DNA nanobrush does significantly improve cellular adhesion efficiency between different cell types.

Next, we investigated the potential of our DNA nanobrush for adhering homogeneous cells to form stable cell clusters and differentiate into microtissue over suitable incubation times. Here, we used a nanobrush (b3-2chol) with a backbone base of 25 bp. As shown in Figure 2B, after 24 h of incubation, the b3-2chol nanobrush regulated CEM cells to form a larger cluster. The median cross-sectional area was determined to be about 7020 μm^2^ by counting 100 clusters, corresponding to 50–150 cells per cluster. Other stability experiments of DNA nanostructure-assembled cell clusters at different times can be found in Figure S6. Live–dead cell staining experiments demonstrated that most cells within the spheroids were still alive after 36 h of incubation (Figure 2C). Besides, we also performed cell clustering using four other nanostructures under prolonged incubation (Figure S7). The result shows that each nanobrush has a good clustering effect, and b3–2chol has the highest assembly efficiency. This is mainly because the b3 backbone is a fully rotated conformation, and therefore has the largest contact area and the highest assembly efficiency.

### DNA Nanobrush-Regulated Intermembrane Distance Determined by MIET Imaging/Spectroscopy

Having established DNA nanobrushes as an effective and reliable nanoglue for plasma membranes that works both between homogeneous cells and heterogeneous cells, we applied MIET imaging/spectroscopy to determine the intermembrane distance, which is modulated by our DNA nanobrushes. A biomimetic membrane system was designed to model cell adhesion between supported lipid bilayers (SLBs, prepared using DOPC (1,2-dioleoyl-*sn*-glycerol-3-phosphocholine)), and fluorescently labeled giant unilamellar vesicles (GUVs, prepared with DOPC and 0.1% DPPE-Atto655 (1,2-Bis(diphenylphosphino)ethane)).

To validate the high spatial resolution of MIET imaging and spectroscopy, a series of DNA nanobrushes with different arm lengths were placed between the SLBs and GUVs. SLBs were prepared on a MIET substrate (10 nm gold film is sandwiched between a coverslip and a 10 nm silica layer, Figure S8) via vesicle fusion. Then, DNA nanobrushes were added and incubated for 30 min to form DNA nanobrush layers on the SLBs (Figure S9). After the unbounded DNA nanobrushes were washed out, fluorescently labeled GUVs were added to the chamber and incubated for another 30 min. The gold film-coated substrate served for inducing a distance-dependent fluorescence lifetime. Fluorescence images and lifetimes of the fluorescently labeled GUVs were taken with a confocal microscope, which was equipped with time-correlated single-photon counting (TCSPC) for fluorescence lifetime measurements.^40^

**Figure 3:**
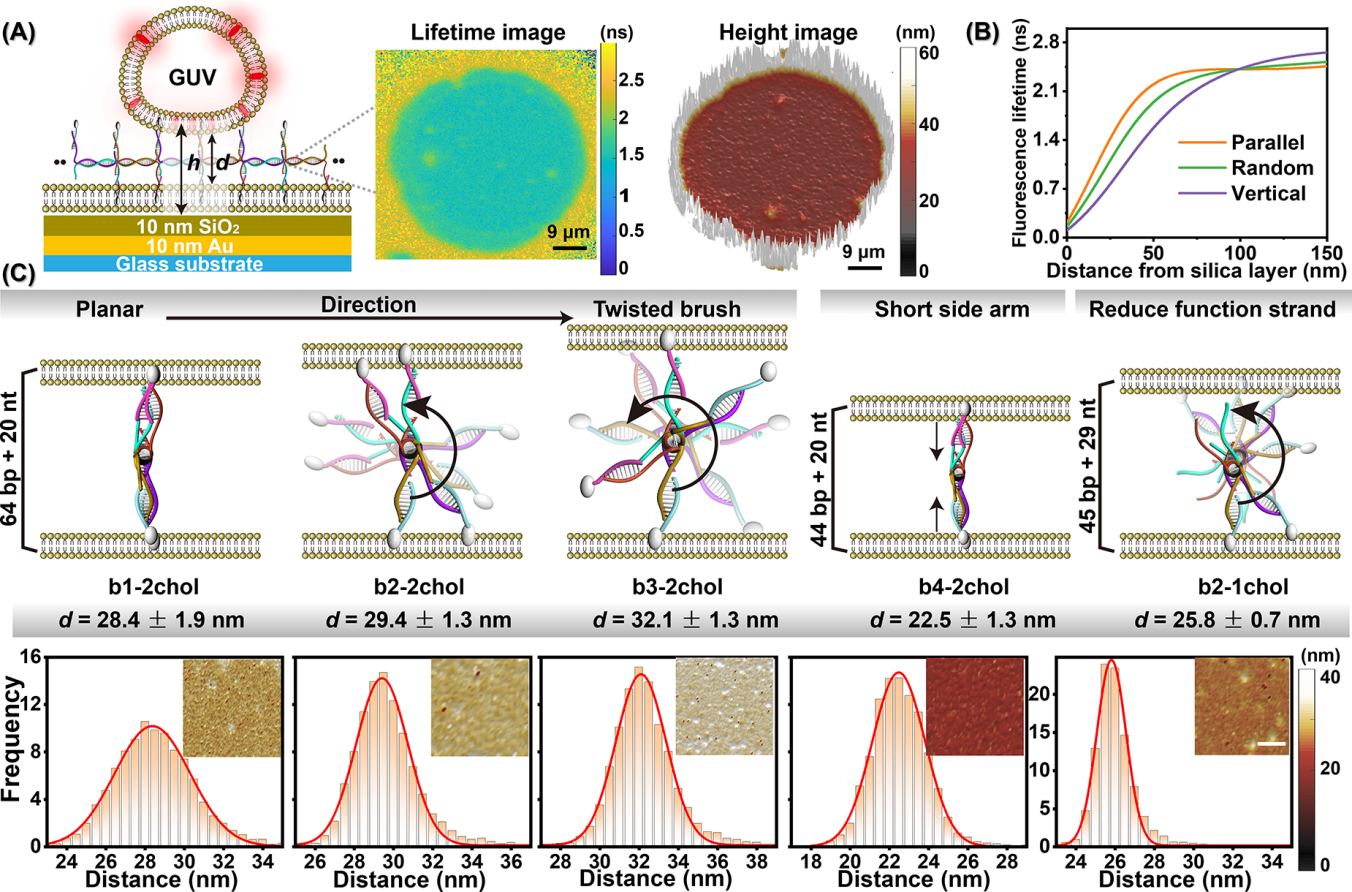
Metal-induced energy transfer (MIET) microscopy to visualize the intermembrane distance regulated by DNA nanobrushes. (A) Schematic illustration of MIET imaging/spectroscopy to measure the distance between a GUV and SLB membranes modulated by DNA nanobrushes. *h* is the height from the center of the proximal membrane of one GUV to the SiO_2_ surface. *d* is the distance between the two membranes. Middle panel: fluorescence lifetime image obtained from calculating the mean lifetime with a maximum likelihood estimation algorithm for each pixel. Right panel: height map *d* of the proximal membrane of a GUV. (B) Calculated dependence of fluorescence lifetime on axial distance from silica surface. Curves were calculated for a dipole emitting at a wavelength of 680 nm and for three different dipole orientations with respect to the interface (vertical, horizontal, random orientation). The MIET substrate was fabricated by depositing 10 nm of gold and 10 nm of SiO_2_ on a cover slide. (C) DNA nanobrushes regulate the average distance *d* between a GUV and a SLB. Red curves show Gaussian fits of the distance distributions. More than 10 GUVs per group were measured, and for each group three independent measurements were performed. Inset: corresponding height map. Scale bar is 9 μm.

We first scanned the sample and found that the proximal membranes of almost all GUVs adhered to the SLBs via DNA nanobrushes. In contrast, GUVs without any DNA brushes did not exhibit adhesion events (Figure S10). To precisely determine the intermembrane distance, we scanned individual GUVs to accumulate signal for fluorescence decay fitting. We constructed TCSPC curves for each pixel and fitted these curves with a multiexponential decay model, giving us a mean fluorescence lifetime for each pixel (Figure S11) .^41^ These lifetime images were then converted to membrane height images above the silica surface using an MIET calibration curve (Figure 3A). A MIET calibration curve was calculated based on a semiclassical electrodynamics model of the near-field coupling between the fluorophore and the substrate, taking into account parameters such as the refractive index of the buffer, the thickness of the metal and silica films, and the quantum yield and emission spectrum of the dye molecules. For the system used here, all the optical parameters for both the dye DPPE-Atto655 (free-space lifetime = 2.60, quantum yield = 0.36) and the Au/SiO_2_ substrate have been published before (more details for calibration curve calculation are given in the Supporting Information) .^28,42^Figure 3B shows three calculated MIET curves for DPPE-Atto655 for three different dye orientations with respect to the 10 nm Au film with a 10 nm spacer. On the basis of the lifetime images, we observed that the proximal membrane shows a uniform height across the supported lipid bilayer (SLB) surface with no height fluctuations. Consistent with our previous findings,^27,43,44^ in the case of a flat planar membrane, we assume a dye orientation parallel to the membrane, with the dipole axis of Atto655 parallel to the surface, which is important for the lifetime-height conversion.

Figure 3A shows a height image measured with the b1-2chol/GUV system. The image displays a uniform attachment of the proximal membrane of the GUVs to the nanobrush. The controllable and uniformly distributed cholesterol-functioned strands on the nanobrush backbone facilitate an even distribution of cholesterol over the membrane surface, preventing the formation of self-aggregating clusters. The bright white color of the GUV’s circumference reflects the substantial distance of its membrane there from the gold surface, which exceeds the measurement range of MIET. For statistical analysis, we quantified the height values or distances of all pixels in the central region and calculated the average values (Figure 3C). The height (*h*) values of the central area were found to be uniform within a range of 30 to 40 nm. Taking the hydration layer of the SLBs (∼2 nm) and the thickness of the lipid bilayer (∼4 nm) into account,^44,45^ the intermembrane distance (*d*_m–m_) was derived by subtracting 8 nm from the height (*h*).

To further evaluate the sensitivity of MIET imaging for detecting subtle changes in DNA nanobrush structure, we repeated experiments for different nanostructures with varying backbone and side arms. We first varied the number of backbone bases (b1; b2; b3) to regulate the arrangement direction of the functional strands, gradually from a planar to a twisted brush. Interestingly, even though the nanobrushes (b1; b2; b3) had the same arm lengths (64 bp +20 nt), the resulting intermembrane distance gradually increases from the planar to the twisted conformation (from 28.4 ± 1.9 nm to 32.1 ± 1.3 nm). This can be attributed to the increasing rigidity of the nanostructure in the rotating conformation, reducing the tilt of the DNA structure between two membranes.^46,47^ Additionally, the distance measured from MIET imaging is closely aligned with the cryo-TEM measurement, which give a mean distance of 27.6 ± 4.8 nm for the b1-2chol DNA nanobrush (Figure S12). Second, we modified the nanobrush by shortening the side arms (b4) for the planar structures. As expected, a reduction of 20 bp in the side arm bases corresponded to a decrease in the distance by 5.9 nm. Theoretically, 20 bps correspond to 6.8 nm in length, and the observed 5.9 reduction suggests an inclination of the planar structure.^48^ In addition, we also changed the number of cholesterol functional strands (b2-1chol). When the number of cholesterol functional strands decreased, the distance decreased accordingly (25.8 ± 0.7 nm). Reducing one side arm results in a decrease of 10 nt. Additionally, the functional side arm transitions from a double-stranded structure to a single-stranded structure, thereby enhancing the flexibility of the nanobrush. This increased flexibility allows the nanobrush to flatten and adhere to the membrane surface, resulting in a shorter intermembrane distance. These results demonstrate that MIET can accurately discern the nanoscale changes in the cell membrane spacing induced by modifications of DNA nanobrushes.

### Real-Time Observation of Cell Surface Engineering by MIET Imaging/Spectroscopy

**Figure 4:**
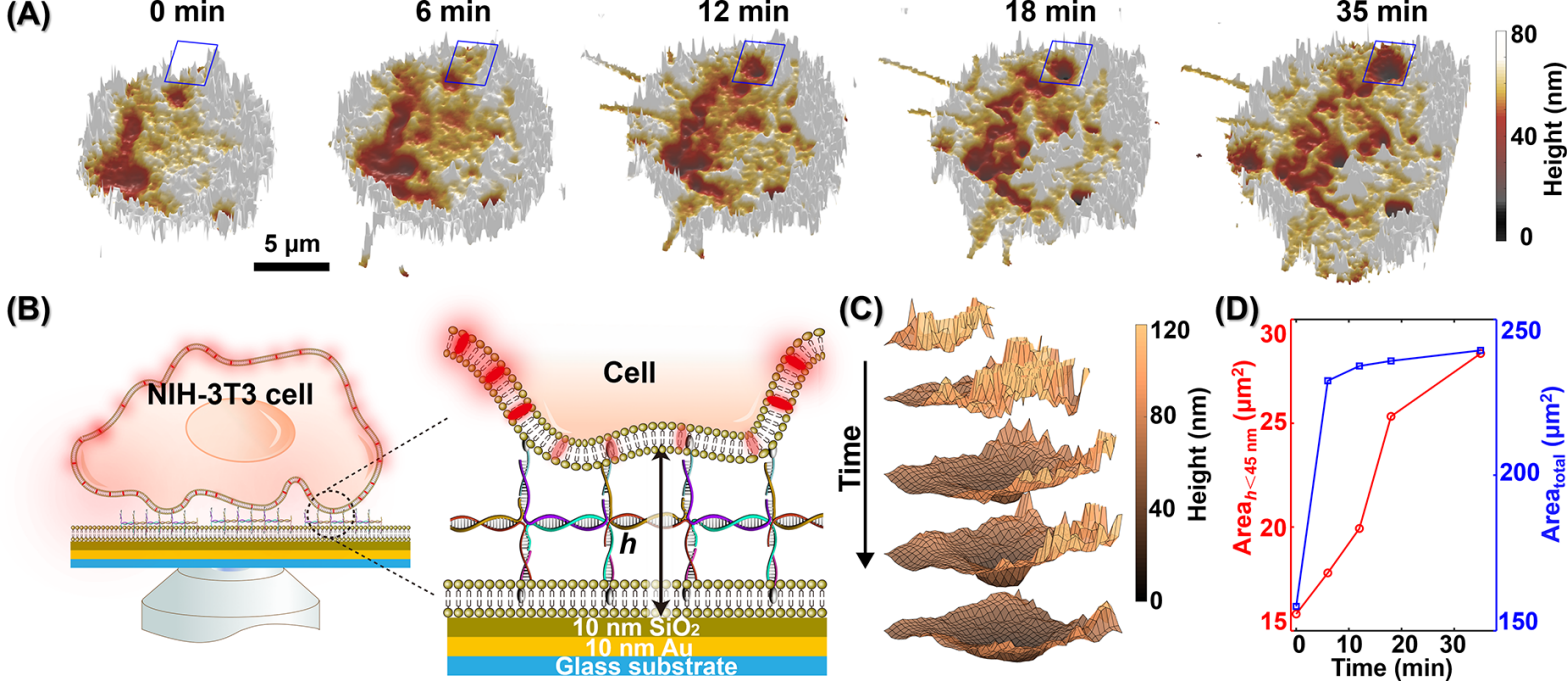
Measurement of DNA-nanobrush regulated binding of a NIH-3T3 cell to an SLB with MIET imaging/spectroscopy. (A) Reconstruction of the 3D height (*h*) maps of the proximal membrane of a NIH-3T3 cell mediated by DNA nanostructures. For each image, the photons are accumulated for 40 s. (B) Schematic illustration of the measurement of a NIH-3T3 cell above SLB with DNA nanobrushes. (C) The enlarged areas are marked in panel A. (D) The statistical analysis of the areas for the contact zones at different times. The red line is the area for the membrane having a height smaller than 45 nm and the blue line is the total area for the whole contact zone.

After demonstrating that MIET imaging/spectroscopy can measure the intermembrane distance between model membranes, we utilized MIET next to monitor distance changes during DNA-nanobrush mediated plasma membrane adhesion of a single cell to a SLB. We replaced GUVs with NIH-3T3 cells (mouse embryonic fibroblast cells) and selected the nanobrush (b1-2chol) as the nanoglue (Figure 4B). The cell’s plasma membrane was labeled by fusing fluorescently labeled fusogenic liposomes with the membrane^49^ or commercial red dye CellMask. Following fluorescent labeling and meticulous washing, our initial step involved determining the height of the basal membrane of NIH-3T3 cells above an MIET substrate devoid of a supported lipid bilayer (SLB). Owing to the influence of the extracellular matrix (ECM) and surface proteins,^50^ the basal membrane maintains an elevated distance of approximately 52 nm from the surface (refer to Figure S13A). Interestingly, this distance falls perfectly within the operational range of MIET, spanning from 5 to 150 nm (Figure 3B). For the adhesion measurement, the cells were added to the DNA-nanobrush modified SLB, which was supported by a Au/SiO_2_ substrate. Once the cell settled onto the SLB, we started to continuously scanning the sample at a scanning rate of 0.4 s/frame over an area of 20 μm × 20 μm. For extracting the dynamics, we obtained TCSPC data for each pixel by frame binning. Then, the fluorescence lifetime values of these pixels were determined with an monoexponential decay model using a maximum likelihood algorithm. Finally, we convert the measured fluorescence lifetime values of each pixel into height values using the MIET curve. Movie S1 shows the height variations over time of one NIH-3T3 cell during adhesion mediated by the DNA nanobrush at a frame rate of 4 s/frame. As the apical cell membrane is at least 500 nm away from the substrate, only dye molecules within the basal membrane were efficiently excited and detected.

For a more precise determination of the height values, we constructed height maps by accumulating one frame over 40 s so that at least 500 photons per pixel contribute to the lifetime calculation. As shown in Figure 4A, the DNA nanobrushes lead to a gradual adhesion of the cell’s plasma membrane to the SLBs. At the beginning of the process, the majority of the membranes are situated at higher elevations with a mean height of ∼70 nm, where only a small fraction with a mean height of ∼40 nm undergoes adhesion. Over time, both the total area of the contact zone and the area of the adhesion zone (height smaller than 45 nm) increased (Figure 4A, D). The increase in the total area may be a consequence of the cell’s extension, and the increased contact area is a direct result of adhesion induced by DNA nanobrushes. A representative enlarged region, as shown in Figure 4C provides a detailed illustration of the adhesion process: the plasma membrane adheres to the SLB, and then the adhesion area expands. Adhesion is probably triggered by membrane fluctuation, resulting in a high probability that membrane patches encounter the cholesterol groups of the DNA nanobrush. After the plasma membrane adheres to the DNA nanobrush, the lowered height of the plasma membrane induces subsequent adhesion of neighboring membrane areas, leading to an extension of the adhesion zone. Finally, we checked that the adhesion of the plasma membrane to the SLB is induced by the DNA nanobrush by conducting control experiments without nanobrush. In the absence of DNA nanobrushes, the 3T3 cells did not adhere to the SLB even after 60 min of incubation (Figure S14). Further, we successfully employed our MIET imaging to monitor the DNA-mediated adhesion processes of other cell lines (COS-7 cells, African green monkey kidney cells; U_2_Os cells, human osteosarcoma cells, Figures S15 and S16), demonstrating the broad applicability of our approach.

## Conclusion

In summary, we have demonstrated the capability of MIET imaging/spectroscopy for monitoring membrane interface changes mediated by DNA nanobrushes. By designing DNA nanobrushes with varying orientation, distance, valence, and flexibility, we used MIET to elucidate subtle changes in membrane spacing as regulated by these DNA nanostructures. Importantly, MIET enables the observation of adhesion as well as adhesion dynamics between the two membranes. Additionally, MIET is straightforward to implement and requires neither any hardware modification of a fluorescence lifetime imaging microscopy (FLIM) system nor the preparation of complex sample substrates. Coating glass cover slides with a thin metal film is the only prerequisite for the technique. It is crucial to highlight that MIET functions within a near-field range, limiting its efficacy in measuring distances between two cells. Despite this constraint, embracing the widely employed SLBs for mimicking biological membranes^4,18,22^ the proficient utilization of MIET to assess intermembrane spacings is beneficial for exploring nanoscale cell surface engineering.

## Experimental Section

### Preparation of DNA Nanobrush

DNA oligonucleotides (Table S1) were synthesized and purified by Sangon Biotech. Co. Ltd. (Shanghai, China). All DNA nanobrushes were synthesized through a “one-pot” process. Briefly, six oligonucleotides with identical molar concentrations were mixed in a 20 mM Tris-HCl buffer (pH 8.0) containing 50 mM MgCl_2_. The mixtures were heated at 95 °C for 10 min and then incubated on ice for 10 min. The as-prepared nanobrushes were stored at 4 °C for further use.

### Nanobrush for Cell Surface Engineering

When Human acute lymphoblastic leukemia CCRF-CEM (abbreviated as CEM) cells and human Burkitt lymphoma Ramos cells were assembled, nanobrush (b1–2chol) at a final concentration of 500 nM were added to ∼10^5^ Ramos cells (prestained with CytoTraceTM Red dye) and incubated for 30 min at 37 °C with a metal bath shaking at 300 rpm. Subsequently, ∼ 10^5^ CEM cells (prestained with CellTracker Green dye) were added and incubated for another 30 min at 37 °C with a metal bath shaking at 300 rpm. The NIKON A1R confocal microscope observed cell assembly with a 490 nm laser and 540 nm laser excitation. Quantitative data of cell assembly were derived by using flow cytometry. For prolonged incubation to build cell clusters, ∼ 10^5^ CEM cells were added nanobrush (b3–2chol) at a final concentration of 500 nM and placed in the incubator at different times. CEM cells will form stable cell clusters within 24 h under the combined effect of hydrophobic insertion. Photographs were taken at different time points using a cell microscope under 10× bright field conditions.

### MIET Instrument and Preparation of MIET Substrate

FLIM measurements were performed using a home-built confocal microscope equipped with a high numerical aperture objective lens (Apo N, 100× oil, 1.49 NA, Olympus Europe, Hamburg, Germany). A pulsed linearly polarized laser (640 nm) with a tunable filter (AOTFnC 400.650-TN, Pegasus Optik GmbH, Wallenhorst, Germany) was used for fluorescence excitation. The light was directed toward the objective through a nonpolarizing beam splitter, and backscattered excitation light was blocked with long-pass filters (FF01–692/40, Semrock). The emission light was focused onto the active area of an avalanche photodiode (PDM Series, MicroPhoton Devices) through a pinhole (100 μm), and the detection times of recorded single photons were determined using a multichannel picosecond event timer (HydraHarp 400, PicoQuant GmbH, Berlin, Germany). A fast Galvo scanner (FLIMbee, Picoquant) was used for imaging scanning. The MIET substrate comprised of a multilayer structure consisting of consecutive layers of 2 nm Ti, 10 nm Au, 1 nm Ti, and 10 nm SiO_2_ on a glass coverslip. This layers were deposited by evaporation using an electron beam source (Univex 350, Leybold) under high-vacuum conditions (∼10^–6^ mbar). Slowest rate of deposition was maintained (1 Å s^–1^) to ensure maximal homogeneity. The spacer thickness was continuously monitored during evaporation with an oscillating quartz unit. This gold-covered substrate is termed the MIET substrate. Characterizations of the AFM and scanning electron microscopy demonstrate the roughness is only 0.8 nm and the MIET substrate exhibits exceptional smoothness and uniformity (see Figure S8).

### Vesicles and SLB Preparation

Small unilamellar vesicles (SUVs) were prepared with an extrusion method. Briefly, 100 μL of 10 mg/mL DOPC lipids in chloroform were dried in a vacuum for 1 h at 30 °C to remove the residual solvent. Then, 500 μL of PBS buffer (pH 7.4) was added, and the solution was shaken for 1 h at 30 °C. The solution was then extruded for 15 cycles through a polycarbonate filter (Whatman) with 50 nm pore diameter. The resulting vesicle solutions were used within 3 days while stored at 4 °C before use. GUVs were fabricated by electroformation^27^ in a custom-built Teflon chamber with two stainless steel electrodes. Briefly, 100 μL of a chloroform solution containing DOPC (10 mg/mL) and 0.1 M Atto655-DPPE was deposited onto two electrodes, followed by evaporation for 3 h under vacuum at 30 °C. The chamber was filled with 500 μL of 300 mM sucrose solution, after which an alternating electric current of 15 Hz frequency and a peak-to-peak voltage of 1.6 V was applied for 3 h, followed by a lower frequency voltage of 8 Hz for another 30 min. Formed GUVs were collected by rinsing the electrode surface with 500 μL of a PBS solution. DOPC SLBs were formed via vesicle fusion. Before placing an SUV solution onto a MIET substrate, the substrate was activated with the plasma of a plasma cleaner (Harrick Plasma, New York, United States) at low intensity for 30 s. Then, a droplet of SUV solution was placed on the substrate and incubated for 1 h to ensure the formation of a uniform bilayer with minimal defects. This was followed by copious washing with buffer.

### Cell Membrane Staining

For cell membrane staining, liposomes were prepared by mixing DOPE/DOTAP/Atto655-DPPE lipids in chloroform in a weight ratio of 1:1:0.1. The chloroform was then evaporated under vacuum for 0.5 h, and the lipids were dispersed in 20 mM HEPES buffer to obtain a final concentration of 2 mg/mL. The solution was vortexed for approximately 2 min to produce multilamellar liposomes. After homogenization in an ultrasonic bath for 20 min, liposomes that were ready for cell membrane staining were obtained. For cell membrane staining experiments, 5 μL of liposome stock solution was diluted 100 times with the appropriate cell culture medium and gently shaken for 1 min at room temperature. Then, ∼10^5^ NIH-3T3 cells were incubated in 500 μL of fusogenic liposome solution (pH 7.4) for 20 min at 37 °C. Subsequently, the cells were washed twice with 500 μL of 1 × PBS buffer and then suspended in 1 mL of fresh medium for future use.

## Supporting Information Available

Reagents, characterization, control experiments, working principle of MIET imaging, table for DNA sequences, schematic diagram of DNA nanobrush, Figures S1–S16 (PDF)
Movie S1, showing the height variations over time of one NIH-3T3 cell during adhesion mediated by the DNA nanobrush at a frame rate of 4 s/frame (AVI)

## Supplementary Material

nn3c10162_si_001.avi

## References

[ref1] Wu J.; Minikes A. M.; Gao M.; Bian H.; Li Y.; Stockwell B. R.; Chen Z.-N.; Jiang X. Intercellular interaction dictates cancer cell ferroptosis via NF2–YAP signalling. Nature 2019, 572, 402–406. 10.1038/s41586-019-1426-6. 31341276 PMC6697195

[ref2] Hui E. E.; Bhatia S. N. Micromechanical control of cell–cell interactions. Proc. Natl. Acad. Sci. U. S. A. 2007, 104, 5722–5726. 10.1073/pnas.0608660104. 17389399 PMC1851558

[ref3] Stevens M. M.; George J. H. Exploring and engineering the cell surface interface. Science 2005, 310, 1135–1138. 10.1126/science.1106587. 16293749

[ref4] Du Y.; Lyu Y.; Lin J.; Ma C.; Zhang Q.; Zhang Y.; Qiu L.; Tan W. Membrane-anchored DNA nanojunctions enable closer antigen-presenting cell–T-cell contact in elevated T-cell receptor triggering. Nat. Nanotechnol. 2023, 18, 818–827. 10.1038/s41565-023-01333-2. 36894782

[ref5] Shi S.; Chen J.; Wang X.; Xiao M.; Chandrasekaran A. R.; Li L.; Yi C.; Pei H. Biointerface Engineering with Nucleic Acid Materials for Biosensing Applications. Adv. Funct. Mater. 2022, 32, 2201069 10.1002/adfm.202270210.

[ref6] Chugh V.; Vijaya Krishna K.; Pandit A. Cell membrane-coated mimics: a methodological approach for fabrication, characterization for therapeutic applications, and challenges for clinical translation. ACS Nano 2021, 15, 17080–17123. 10.1021/acsnano.1c03800. 34699181 PMC8613911

[ref7] Wang D.-X.; Wang J.; Wang Y.-X.; Du Y.-C.; Huang Y.; Tang A.-N.; Cui Y.-X.; Kong D.-M. DNA nanostructure-based nucleic acid probes: construction and biological applications. Chem. Sci. 2021, 12, 7602–7622. 10.1039/D1SC00587A. 34168817 PMC8188511

[ref8] Chen L.; Chen W.; Liu G.; Li J.; Lu C.; Li J.; Tan W.; Yang H. Nucleic acid-based molecular computation heads towards cellular applications. Chem. Soc. Rev. 2021, 50, 12551–12575. 10.1039/D0CS01508C. 34604889

[ref9] Feng L.; Li J.; Sun J.; Wang L.; Fan C.; Shen J. Recent Advances of DNA Nanostructure-Based Cell Membrane Engineering. Adv. Healthcare Mater. 2021, 10, 2001718 10.1002/adhm.202001718. 33458966

[ref10] Adebowale K.; Liao R.; Suja V. C.; Kapate N.; Lu A.; Gao Y.; Mitragotri S. Materials for Cell Surface Engineering. Adv. Mater. 2023, 2210059 10.1002/adma.202210059. 36809574

[ref11] Ge Z.; Liu J.; Guo L.; Yao G.; Li Q.; Wang L.; Li J.; Fan C. Programming cell–cell communications with engineered cell origami clusters. J. Am. Chem. Soc. 2020, 142, 8800–8808. 10.1021/jacs.0c01580. 32302107

[ref12] Shi C.; Zhang Q.; Yao Y.; Zeng F.; Du C.; Nijiati S.; Wen X.; Zhang X.; Yang H.; Chen H.; et al. Targeting the activity of T cells by membrane surface redox regulation for cancer theranostics. Nat.Nanotechnol. 2023, 18, 86–97. 10.1038/s41565-022-01261-7. 36536041

[ref13] Li J.; Xun K.; Pei K.; Liu X.; Peng X.; Du Y.; Qiu L.; Tan W. Cell-membrane-anchored DNA nanoplatform for programming cellular interactions. J. Am. Chem. Soc. 2019, 141, 18013–18020. 10.1021/jacs.9b04725. 31626550

[ref14] Gao F.; Yang D.; Xu F.; Ma X.; Wang P. Promoting Cell Fusion by Polyvalent DNA Ligands. Nano Lett. 2022, 22, 3018–3025. 10.1021/acs.nanolett.2c00216. 35362981

[ref15] Stevens A. J.; Harris A. R.; Gerdts J.; Kim K. H.; Trentesaux C.; Ramirez J. T.; McKeithan W. L.; Fattahi F.; Klein O. D.; Fletcher D. A.; et al. Programming multicellular assembly with synthetic cell adhesion molecules. Nature 2023, 614, 144–152. 10.1038/s41586-022-05622-z. 36509107 PMC9892004

[ref16] Engel B. D.; Schaffer M.; Kuhn Cuellar L.; Villa E.; Plitzko J. M.; Baumeister W. Native architecture of the Chlamydomonas chloroplast revealed by in situ cryo-electron tomography. elife 2015, 4, e04889 10.7554/eLife.04889. 25584625 PMC4292175

[ref17] Blum T. B.; Hahn A.; Meier T.; Davies K. M.; Kühlbrandt W. Dimers of mitochondrial ATP synthase induce membrane curvature and self-assemble into rows. Proc. Natl. Acad. Sci. U. S. A. 2019, 116, 4250–4255. 10.1073/pnas.1816556116. 30760595 PMC6410833

[ref18] Armanious A.; Gerelli Y.; Micciulla S.; Pace H. P.; Welbourn R. J.; Sjöberg M.; Agnarsson B.; Höök F. Probing the Separation Distance between Biological Nanoparticles and Cell Membrane Mimics Using Neutron Reflectometry with Sub-Nanometer Accuracy. J. Am. Chem. Soc. 2022, 144, 20726–20738. 10.1021/jacs.2c08456. 36326176 PMC9673153

[ref19] Wang C.; Taki M.; Sato Y.; Tamura Y.; Yaginuma H.; Okada Y.; Yamaguchi S. A photostable fluorescent marker for the superresolution live imaging of the dynamic structure of the mitochondrial cristae. Proc. Natl. Acad. Sci. U. S. A. 2019, 116, 15817–15822. 10.1073/pnas.1905924116. 31337683 PMC6689947

[ref20] Khalili A.; Ahmad M. A review of cell adhesion studies for biomedical and biological applications. Int. J. Mol. Sci. 2015, 16, 18149–18184. 10.3390/ijms160818149. 26251901 PMC4581240

[ref21] Schmidt D.; Monzel C.; Bihr T.; Merkel R.; Seifert U.; Sengupta K.; Smith A.-S. Signature of a nonharmonic potential as revealed from a consistent shape and fluctuation analysis of an adherent membrane. Phys. Rev. X 2014, 4, 021023 10.1103/PhysRevX.4.021023.

[ref22] Fenz S. F.; Bihr T.; Schmidt D.; Merkel R.; Seifert U.; Sengupta K.; Smith A.-S. Membrane fluctuations mediate lateral interaction between cadherin bonds. Nat. Phys. 2017, 13, 906–913. 10.1038/nphys4138.

[ref23] Ma D.-F.; Xu C.-H.; Hou W.-Q.; Zhao C.-Y.; Ma J.-B.; Huang X.-Y.; Jia Q.; Ma L.; Diao J.; Liu C.; et al. Detecting Single-Molecule Dynamics on Lipid Membranes with Quenchers-in-a-Liposome FRET. Angew. Chem., Int. Ed. 2019, 58, 5577–5581. 10.1002/anie.201813888. 30838761

[ref24] Hou W.; Ma D.; He X.; Han W.; Ma J.; Wang H.; Xu C.; Xie R.; Fan Q.; Ye F.; et al. Subnanometer-precision measurements of transmembrane motions of biomolecules in plasma membranes using quenchers in extracellular environment. Nano Lett. 2021, 21, 485–491. 10.1021/acs.nanolett.0c03941. 33280386

[ref25] Li Y.; Qian Z.; Ma L.; Hu S.; Nong D.; Xu C.; Ye F.; Lu Y.; Wei G.; Li M. Single-molecule visualization of dynamic transitions of pore-forming peptides among multiple transmembrane positions. Nat. commun. 2016, 7, 12906 10.1038/ncomms12906. 27686409 PMC5056435

[ref26] Chizhik A. I.; Rother J.; Gregor I.; Janshoff A.; Enderlein J. Metal-induced energy transfer for live cell nanoscopy. Nat. Photonics 2014, 8, 124–127. 10.1038/nphoton.2013.345.

[ref27] Ghosh A.; Sharma A.; Chizhik A. I.; Isbaner S.; Ruhlandt D.; Tsukanov R.; Gregor I.; Karedla N.; Enderlein J. Graphene-based metal-induced energy transfer for sub-nanometre optical localization. Nat. Photonics 2019, 13, 860–865. 10.1038/s41566-019-0510-7.

[ref28] Niehörster T.; Löschberger A.; Gregor I.; Krämer B.; Rahn H.-J.; Patting M.; Koberling F.; Enderlein J.; Sauer M. Multi-target spectrally resolved fluorescence lifetime imaging microscopy. Nat. methods 2016, 13, 257–262. 10.1038/nmeth.3740. 26808668

[ref29] Chizhik A. M.; Ruhlandt D.; Pfaff J.; Karedla N.; Chizhik A. I.; Gregor I.; Kehlenbach R. H.; Enderlein J. Three-dimensional reconstruction of nuclear envelope architecture using dual-color metal-induced energy transfer imaging. ACS Nano 2017, 11, 11839–11846. 10.1021/acsnano.7b04671. 28921961

[ref30] Raja S. O.; Chizhik A. I.; Schmidt C. F.; Enderlein J.; Ghosh A. Mapping activity-dependent quasi-stationary states of mitochondrial membranes with graphene-induced energy transfer imaging. Nano Lett. 2021, 21, 8244–8249. 10.1021/acs.nanolett.1c02672. 34520214

[ref31] Duckett D. R.; Murchie A. I.; Diekmann S.; von Kitzing E.; Kemper B.; Lilley D. M. The structure of the Holliday junction, and its resolution. Cell 1988, 55, 79–89. 10.1016/0092-8674(88)90011-6. 3167979

[ref32] McKinney S. A.; Déclais A.-C.; Lilley D. M.; Ha T. Structural dynamics of individual Holliday junctions. Nat. Struct. Biol. 2003, 10, 93–97. 10.1038/nsb883. 12496933

[ref33] Ke Y.; Douglas S. M.; Liu M.; Sharma J.; Cheng A.; Leung A.; Liu Y.; Shih W. M.; Yan H. Multilayer DNA origami packed on a square lattice. J. Am. Chem. Soc. 2009, 131, 15903–15908. 10.1021/ja906381y. 19807088 PMC2821935

[ref34] Wang J. C. Helical repeat of DNA in solution. Proc. Natl. Acad. Sci. U. S. A. 1979, 76, 200–203. 10.1073/pnas.76.1.200. 284332 PMC382905

[ref35] Yoshihara A.; Watanabe S.; Goel I.; Ishihara K.; Ekdahl K. N.; Nilsson B.; Teramura Y. Promotion of cell membrane fusion by cell-cell attachment through cell surface modification with functional peptide-PEG-lipids. Biomaterials 2020, 253, 120113 10.1016/j.biomaterials.2020.120113. 32438114

[ref36] Qian R.-C.; Zhou Z.-R.; Guo W.; Wu Y.; Yang Z.; Lu Y. Cell surface engineering using DNAzymes: metal ion mediated control of cell–cell interactions. J. Am. Chem. Soc. 2021, 143, 5737–5744. 10.1021/jacs.1c00060. 33749281 PMC9170360

[ref37] Ke Y.; Ong L. L.; Shih W. M.; Yin P. Three-dimensional structures self-assembled from DNA bricks. science 2012, 338, 1177–1183. 10.1126/science.1227268. 23197527 PMC3843647

[ref38] Iinuma R.; Ke Y.; Jungmann R.; Schlichthaerle T.; Woehrstein J. B.; Yin P. Polyhedra self-assembled from DNA tripods and characterized with 3D DNA-PAINT. science 2014, 344, 65–69. 10.1126/science.1250944. 24625926 PMC4153385

[ref39] McKinnon K. M. Flow cytometry: an overview. Curr. Protoc. Immunol. 2018, 120, 10.1002/cpim.40. PMC5939936 29512141

[ref40] Kapusta P.; Wahl M.; Benda A.; Hof M.; Enderlein J. Fluorescence lifetime correlation spectroscopy. J. Fluoresc. 2006, 17, 43–48. 10.1007/s10895-006-0145-1. 17171439

[ref41] Thiele J. C.; Helmerich D. A.; Oleksiievets N.; Tsukanov R.; Butkevich E.; Sauer M.; Nevskyi O.; Enderlein J. Confocal fluorescence-lifetime single-molecule localization microscopy. ACS Nano 2020, 14, 14190–14200. 10.1021/acsnano.0c07322. 33035050

[ref42] Zelená A.; Isbaner S.; Ruhlandt D.; Chizhik A.; Cassini C.; Klymchenko A. S.; Enderlein J.; Chizhik A.; Köster S. Time-resolved MIET measurements of blood platelet spreading and adhesion. Nanoscale 2020, 12, 21306–21315. 10.1039/D0NR05611A. 33073832

[ref43] Ghosh A.; Chizhik A. I.; Karedla N.; Enderlein J. Graphene-and metal-induced energy transfer for single-molecule imaging and live-cell nanoscopy with (sub)-nanometer axial resolution. Nat. Protocols 2021, 16, 3695–3715. 10.1038/s41596-021-00558-6. 34099942

[ref44] Chen T.; Ghosh A.; Enderlein J. Cholesterol-induced nanoscale variations in the thickness of phospholipid membranes. Nano Lett. 2023, 23, 2421–2426. 10.1021/acs.nanolett.2c04635. 36706024 PMC10037415

[ref45] Tero R. Substrate effects on the formation process, structure and physicochemical properties of supported lipid bilayers. Materials 2012, 5, 2658–2680. 10.3390/ma5122658.

[ref46] Pan M.; Shi J.; Wang L.; Fan C.; Liu X. Cryogenic Electron Microscopy for Resolving DNA Nanostructures and Their Complexes. Small Struct 2021, 2, 2100053 10.1002/sstr.202100053.

[ref47] Wan N.; Hong Z.; Wang H.; Fu X.; Zhang Z.; Li C.; Xia H.; Fang Y.; Li M.; Zhan Y.; Yang X.; et al. A DNA origami mechanical device for the regulation of microcosmic structural rigidity. small 2017, 13, 1700866 10.1002/smll.201700866. 28902974

[ref48] Castro C. E.; Kilchherr F.; Kim D.-N.; Shiao E. L.; Wauer T.; Wortmann P.; Bathe M.; Dietz H. A primer to scaffolded DNA origami. Nat. Methods 2011, 8, 221–229. 10.1038/nmeth.1570. 21358626

[ref49] Csiszár A.; Hersch N.; Dieluweit S.; Biehl R.; Merkel R.; Hoffmann B. Novel fusogenic liposomes for fluorescent cell labeling and membrane modification. Bioconjugate Chem. 2010, 21, 537–543. 10.1021/bc900470y. 20184308

[ref50] Yue B. Biology of the extracellular matrix: an overview. J. Glaucoma 2014, 23, S20 10.1097/IJG.0000000000000108. 25275899 PMC4185430

