# Supplementary material for: Metal-Induced Energy Transfer (MIET) Imaging of Cell Surface Engineering with Multivalent DNA Nanobrushes
Source: ACS Nano. 2024 Jan 17;18(7):5409–17. doi: 10.1021/acsnano.3c10162 (PMC10883130; doi:10.1021/acsnano.3c10162)
Supplement: Supplementary file 2 — nn3c10162_si_002.pdf [file nn3c10162_si_002.pdf]

Supporting Information

Metal-induced energy transfer (MIET) imaging of cell surface engineering with multivalent DNA nanobrushes

Dong-Xia Wang,[1,2] Bo Liu,[1] Gui-Mei Han,[1] Qingnan Li,[1] De-Ming Kong,[1,*] Jörg Enderlein,[2,3,*] Tao Chen[2,*]

[1]State Key Laboratory of Medicinal Chemical Biology, Tianjin Key Laboratory of Biosensing and Molecular Recognition, Research Centre for Analytical Sciences, College of Chemistry, Nankai University, Tianjin 300071, P. R. China

[2] III. Institute of Physics - Biophysics, Georg August University, 37077 Göttingen, Germany

[3]Cluster of Excellence "Multiscale Bioimaging: from Molecular Machines to Networks of Excitable Cells" (MBExC), Universitätsmedizin Göttingen, Robert-Koch-Str. 40, 37075 Göttingen, Germany

Table of Contents

Experimental section…..................................…………………………………......………………………........................ S-3
Schematic diagram of DNA nanobrush………………………………………...S-8
Synthesis and characterization of the DNA nanobrush.......................………………...S-11
Optimization of two kinds of cells assembled by nanobrush.................……………………………...........................S-13
Stability of assembly of CCRF-CEM cells and nanobrush under long-term incubation…………………...................S-15
CCRF-CEM cells assembled into clusters with different nanobrush........…………....…………..………………...........S-16
Characterize the smoothness and uniformity of MIET substrate surface.......………………….....................................S-17
Scheme of MIET experimental set-up…………………………….........……...S-18
MIET measurements of DNA nanobrush-assembled membranes...........…………………….......................................S-19
Cryo-TEM to visualize the inter-membrane distance regulated by DNA nanobrush.......……………........................S-21
MIET microscopy to visualize binding process using NIH-3T3 cells.........……………………..........…........................S-22
MIET microscopy to visualize the binding process using COS-7 cells......……………...S-24
MIET microscopy to visualize the binding process using U_2Os cells.......…………………...S-25
References…………………………...S-26

Video supporting information

Video S1: Height variations of one NIH-3T3 cell during the adhesion process mediated by the DNA nanobrush.

Experimental Procedures

Materials and reagents

All DNA oligonucleotides (Table S1) were synthesized and purified by Sangon Biotech. Co. Ltd. (Shanghai, China). CM-DiO (green), CM-DiI (red), CellTracker Green CMFDA (green), and CytoTraceTM Red Fluorescent Probe (red) were purchased from Yeasen (Shanghai, China). Calcein-AM and propidium iodide (PI) were obtained from Meilunbio (Dalian, China). Fetal bovine serum (FBS), Roswell Park Memorial Institute (RPMI) 1640, and Dulbecco's modified Eagle's medium (DMEM) were purchased from Gibco (Grand Island, NY, USA). 1,2-dioleoyl-sn-glycero-3-phosphatidylcholine (DOPC), 1,2-dioleoyl-3-trimethylammonium-propane, chloride salt (DOTAP), and atto655 labled 1,2-Bis(diphenylphosphino)ethan (atto655-DPPE) were purchased from Sigma-Aldich (Germany). CellMask™ Deep Red Stain were obtained from Thermo Fisher (Germany). All other reagents were analytical grade and used directly without further purification. Ultrapure water (resistance ≥ 18.2 MΩ·cm) was used throughout the experiments.

Apparatus

The cell fluorescence images were captured using a confocal laser scanning microscope (Nikon A1R) equipped with 4×, 20×, 60× and 100× objective lenses. All MIET measurements were carried out with a homebuilt confocal microscope equipped with a multichannel picosecond event timer (HydraHarp 400, PicoQuant GmbH) allowing for fluorescence lifetime imaging. Atomic force microscopy (AFM) characterization was carried out using a Bruker Dimension Icon (USA). Flow cytometry results were recorded on a FACS Calibur (BD, USA). Scanning electron microscope (SEM) images were measured on JEOL JSM-7500F (Japan). Cryo-TEM data were acquired using a spherical aberration (Cs) corrected FEI Titan Krios (Thermo Fisher Scientific) transmission electron microscope.

Table S1. Sequences of the oligonucleotides used in this work.

Oligonucleotide		Sequences (5' to 3')
**Nanobrush backbone 21 bases (b1)**	S1-21	GAAAGAAACAACCCTTGCGCATTCGAGTCTCCACATTTAGTTCTCATCCTTTCCATTCGAGTCTCC
	S2-21	TATTGTTTCTTGGTAGTCTTTAGCTGCAGTGTGAAAGGATGAGAACTAAATGTTTAGCTGCAGTGT
	S3-21	GAAAGAAACAACCCTTGCGGGAGACTCGAATGTAGTATCCCACATTCTCTTTCGGAGACTCGAATG
	S4-21	TATTGTTTCTTGGTAGTCTACACTGCAGCTAAGAAAGAGAATGTGGGATACTAACACTGCAGCTAA
**Nanobrush backbone 22 bases (b2)**	S1-22	GAAAGAAACAACCCTTGCGCATTCGAGTCTCCCACATTTAGTTCTCATCCTTTCCATTCGAGTCTCC
	S2-22	TATTGTTTCTTGGTAGTCTTTAGCTGCAGTGTGAAAGGATGAGAACTAAATGTGTTAGCTGCAGTGT
	S3-22	GAAAGAAACAACCCTTGCGGGAGACTCGAATGTTAGTATCCCACATTCTCTTTCGGAGACTCGAATG

	S4-22	TATTGTTTCTTGGTAGTCTACACTGCAGCTAAGAAAGAGAATGTGGGATACTAAACACTGCAGCTAA
Nanobrush backbone 25 bases (b3)	S1-25	GAAAGAAACAACCCTTGCGCATTCGAGTCTCCCACATTTAGTGCGTCTCATCCTTTCCATTCGAGTCTCC
	S2-25	TATTGTTTCTTGGTAGTCTTTAGCTGCAGTGTGAAAGGATGAGACGCACTAAATGTGTTAGCTGCAGTGT
	S3-25	GAAAGAAACAACCCTTGCGGGAGACTCGAATGTTAGTATCCCATGCCATTCTCTTTCGGAGACTCGAATG
	S4-25	TATTGTTTCTTGGTAGTCTACACTGCAGCTAAGAAAGAGAATGGCATGGGATACTAAACACTGCAGCTAA
Nanobrush backbone 21 bases short side arm (b4)	s-S1-21	ACCCTTGCGCATTCGAGTCTCCACATTTAGTTCTCATCCTTTCCATTCGAGTCTCC
	s-S2-21	TGGTAGTCTTTAGCTGCAGTGTGAAAGGATGAGAACTAAATGTTTAGCTGCAGTGT
	s-S3-21	ACCCTTGCGGGAGACTCGAATGTAGTATCCCACATTCTCTTTCGGAGACTCGAATG
	s-S4-21	TGGTAGTCTACACTGCAGCTAAGAAAGAGAATGTGGGATACTAACACTGCAGCTAA
Functional strand	chol-1	CGCAAGGGTTGTTTCTTTCTTTATCTAAC-chol
	chol-1-FAM	FAM-CGCAAGGGTTGTTTCTTTCTTTATCTAAC-chol
	s-chol-1	CGCAAGGGTTTTATCTAAC-chol
	chol-2	AGACTACCAAGAAACAATATTTATCTAAC-chol
	s-chol-2	AGACTACCATTTATCTAAC-chol
	2chol-ssDNA	chol-CGCAAGGGTTGTTTCTTTCTTTATCTAAC-chol

The green and yellow parts represent the backbone of the nanobrush, with S1 and S2 being complementary, and S3 and S4 being complementary as well. The side arms are depicted in red and blue parts, respectively.

Preparation and characterization of DNA Nanobrush

All DNA nanobrushes were synthesized through a "one-pot" process. Briefly, six oligonucleotides (Table S1) with identical molar concentrations were mixed in 20 mM Tris-HCl buffer (pH 8.0) containing 50 mM $MgCl_2$. The mixtures were heated at 95 ^{o}C for 10 min and then incubated on ice for 10 min. The as-prepared nanobrushes were stored at 4 °C for further use. The successful synthesis of the above DNA nanobrushes was validated through polyacrylamide gel electrophoresis (PAGE) gel. 2 μL 6 × Super GelRed Prestain Loading Buffer (US EVERBRIGHT INC.) was added to the above samples and sufficiently mixed. The samples were analyzed by 10% PAGE in 1 × TAE buffer at a constant voltage of 120 V for 50 min. The gel was visualized using a Gel Image System (Amersham Biosciences).

The successful synthesis of nanobrush was also analyzed using atomic force microscopy (AFM). To do this, 1 μM DNA nanobrush samples in a total volume of 50 μL were deposited onto a fresh mica surface. The samples were allowed to adsorb for 15 minutes. After that, 30 μL of ultrapure water was added to the sample and allowed to stay on the surface

of the mica sheet for 1 minute. The sample was then absorbed with filter paper, and the washing step was repeated more than 10 times. Finally, the sample was dried with compressed air. The samples were imaged by atomic force microscopy in air scan mode.

Multivalent and monovalent nanobrush binding CEM cells

For monovalent cholesterol-binding CEM cells, 1×10^5 CEM cells were seeded 24 h in advance. After centrifugation to remove the complete medium, 500 μL of 1 × PBS buffer and 100 nM cholesterol-FAM were added and the cells were incubated for 15 minutes at 37 °C. The cells were washed twice with 500 μL of 1 × PBS buffer and then suspended in 1 mL of PBS buffer. To achieve multivalent cholesterol-binding CEM cells, the cholesterol-FAM was simply replaced with nanobrush-FAM. Fluorescence microscopy (Nikon A1R) was used for cell imaging, with the laser excitation wavelength set at 490 nm for FAM. The cell images were observed using a 100× oil objective lens.

Planar nanobrush for two kinds of cell assembly

Human acute lymphoblastic leukemia CCRF-CEM (abbreviated as CEM) cells and human Burkitt lymphoma Ramos cells were cultured in RPMI 1640 medium (GIBCO). The medium was supplemented with 10% fetal bovine serum (FBS) and 1% antibiotics (penicillin-streptomycin-amphotericin B) and the cells were incubated at 37 °C in a CO_2 incubator. 1×10^6 Ramos cells were mixed in 5 mL of 1640 medium, puted into a culture flask, and cultured overnight for 12 hours. Then, 1 mL of Ramos cells were taken and centrifuged at 1200 rpm for 3 min. The cells were resuspended with 100 μL PBS and stained with CytoTraceTM Red Fluorescent Probe (red). The cells were then adjusted to 1×10^5 for future use. Similarly, for CEM cells, 1×10^5 cells were stained with CellTracker Green CMFDA (green) and used for subsequent experiments.

When the two kinds of cells were assembled, b1-2chol at a final concentration of 500 nM was added to 1×10^5 Ramos cells and incubated for 30 min at 37 °C with a metal bath shaking at 300 rpm. The nanobrush was first anchored to the Ramos cell membrane surface by the hydrophobic effect of cholesterol. Subsequently, 1×10^5 CEM cells were added and incubated for another 30 min at 37 °C with a metal bath shaking at 300 rpm. The nanobrush is again anchored to the CEM cell membrane surface by the hydrophobic effect of cholesterol, and finally, the cell assembly is obtained. The NIKON A1R confocal microscope observed cell assembly with 490 nm laser and 540 nm laser excitation. The cell images were observed with 100× objective lens. Quantitative data of cell assembly were derived using flow cytometry with 490 nm channel and 540 nm channel excitation. The above operation was repeated 3 times.

Twisted DNA nanobrush for prolonged incubation to build cell clusters

Individual CEM cells to form cell clusters: 1×10^6 CEM cells were mixed in 5 mL of 1640 medium, placed in a culture bottle and cultured overnight for 12 h. Among them, 1 mL of CEM cells was taken and centrifuged at 1200 rpm for 3 min. We resuspended the cell in 200 μL of 1640 medium and adjusted the cells to 1×10^5 for subsequent use. Then CEM cells were added nanobrush (b3-2chol) at a final concentration of 500 nM and placed in the incubator at different times. CEM cells will form stable cell clusters within 24 hours under the combined effect of hydrophobic insertion. Photographs were taken at different time points using a cell microscope under bright field conditions. We shook the culture flask before imaging to prevent the aggregation of cells during prolonged culture. The magnification of the microscope was 10×.

Working principle of MIET and lifetime-distance conversion

The principle of MIET has been elaborated in our previous publications (ref 1-5). Similar to the fluorescence resonance energy transfer (FRET) process, the gold film can work as an efficient energy acceptor of the excited state energy of a fluorophore. This leads to a strongly distance-dependent modulation of lifetime of the fluorophore over a distance range of ~ 150 nm above the gold surface. This modulation can be calculated by modeling the emitting fluorescent molecule as an electric dipole emitter and solving Maxwell's equation with this source field in the presence of the MIET substrate. Taking into account also the non-radiative transition rate, the observable excited-state fluorescence lifetime (τ_f) is then found as

$$\frac{\tau_f(\theta, z_0)}{\tau_0} = \frac{S_0}{\phi S(\theta, z_0) + (1-\phi)S_0}$$

where $S(\theta, z_0)$ is the emission power of the dipole emitter at the distance of z_0 from the substrate surface with orientation angle of θ, τ_0 is the free-space fluorescence lifetime in absence of the gold film, φ represents the quantum yield of the fluorophore, and S_0 is the free-space emission power of an ideal electric dipole emitter given $S_0 = cnk_0{}^4p^2/3$ with c being the speed of light, k_0 the wave vector amplitude in vacuum, n the refractive index of water, and p the amplitude of emission dipole moment vector. MIET exploits this lifetime-to distance (τ_f versus z) dependence for converting measured lifetime values into distance values, see Figure 3 in main text. To calculate this model curve, *a priori* knowledge of the fluorophore's φ, τ_0, and θ is required. Previously, we have determined these values for DPPE-atto655: $\varphi = 0.36$, $\tau_0 = 2.6$ ns. The fluorophore orientation we used for the GUVs measurement is parallel to the membrane and for the cell measurement we used a random orientation.

For the conversion of fluorescence lifetimes into distance values, we used the calculated lifetime-versus-distance MIET curve as described above (see also Figure 3 in main text). For this purpose, a custom-written MATLAB script was used. A MATLAB-based software package for the calculation of MIET lifetime-versus-distance curves as well as the conversion of a lifetime to distance, equipped with a graphical user interface, has been published (ref 6) and is available free of charge at https://projects.gwdg.de/projects/miet. While the published version of the software assumes that the fluorescent emitters are rotating quickly compared to their excited-state lifetime, this was not the case for the measurements in the present work. Here, a dye orientation parallel to the bilayer (and thus to the substrate) was assumed when calculating the MIET calibration curve.

Inter-membrane distance measurement of model membrane

For MIET measurement, 10 μL of 1 μM nanobrushes were added to the SLBs and incubated for 30 min, followed washing by copious buffer to remove the unbonded nanostructures. The diluted GUVs were added onto the nanostructures and incubated for 30 min.

Real-time observation of cell surface engineering process by MIET

Mouse embryonic fibroblast cells (NIH-3T3 cell), human osteosarcoma cells (U_2Os cells), and African green monkey kidney cells (COS-7 cells) were cultured in DMEM medium (GIBCO), which was supplemented with 10% fetal bovine serum (FBS) and 1% antibiotics (penicillin-streptomycin-amphotericin B) at 37 °C in a CO_2 incubator.

For real-time observation of cells, similar to the GUV measurement, SUVs were deposited on the MIET substrate and fused at room temperature for 30 min to form a uniform lipid bilayer. The substrate was then washed with copious PBS buffer to remove unbound vesicles. Next, 10 μL of 1 μM nanobrush was added to the SLBs and incubated for 30 minutes. After incubation, the substrate was washed with PBS buffer to remove the unbounded nanobrush. Liposome-

stained NIH-3T3 cells were then added to the system. Once the cell settled onto the SLB, continuous scanning of the sample was started. A stage top incubator (Cat. No: 12722, Silver Line, ibidi) fits in the microscope, connects to incubator temperature, gas and humidity controllers and creates the proper environment (37 °C, 5% CO_2) for live-cell imaging right on the microscope stage.

Results and Discussion

Schematic diagram of DNA nanobrush

(A) DNA nanobrush with backbone 21 bp (b1)

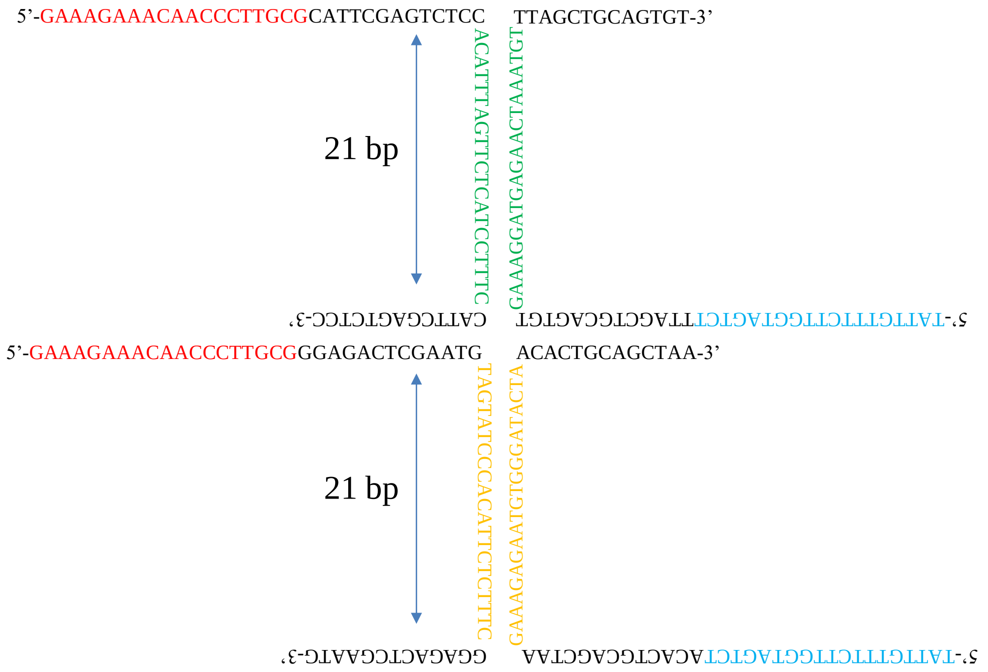

(B) DNA nanobrush with backbone 21 bp ligated cholesterol (b1-2chol)

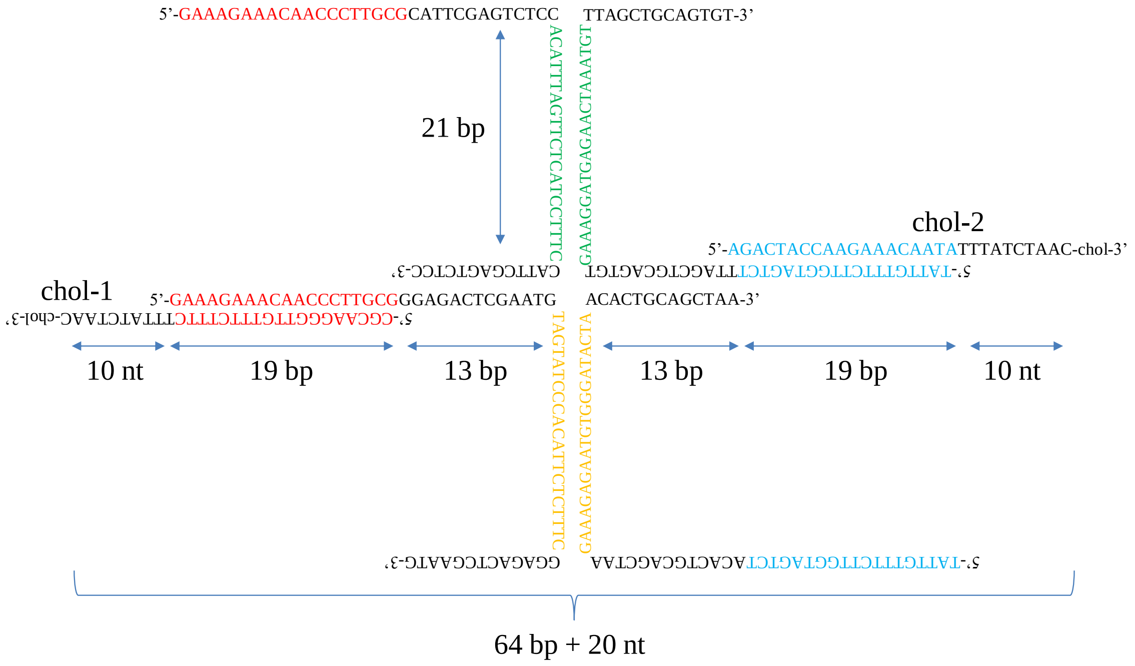

(C) DNA nanobrush with backbone 22 bp ligated cholesterol (b2-2chol)

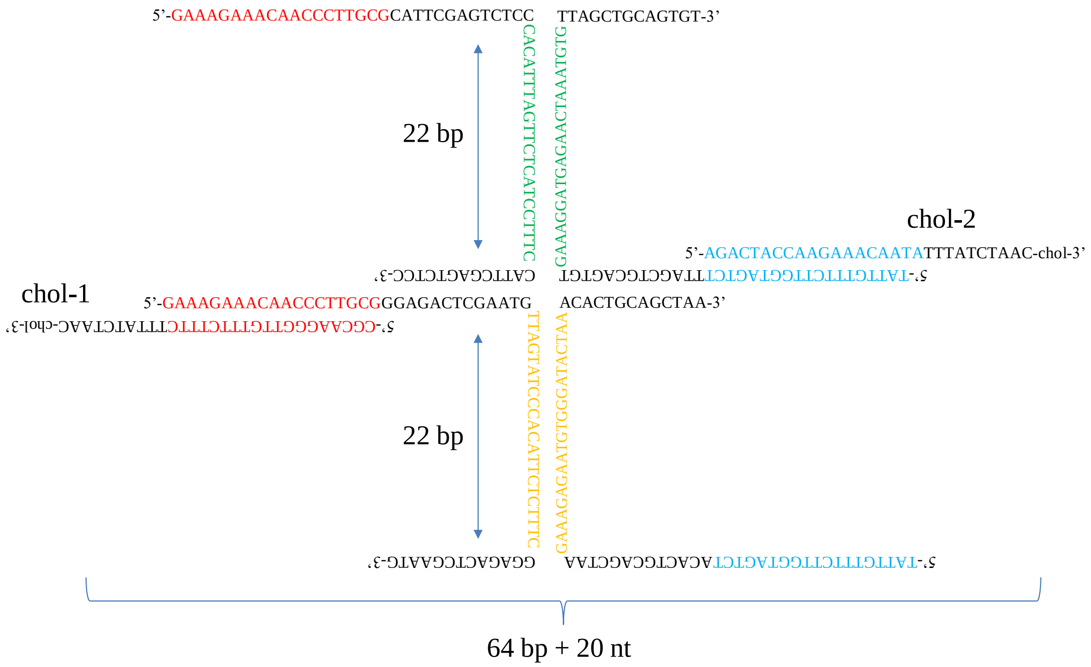

(D) DNA nanobrush with backbone 25 bp ligated cholesterol (b3-2chol)

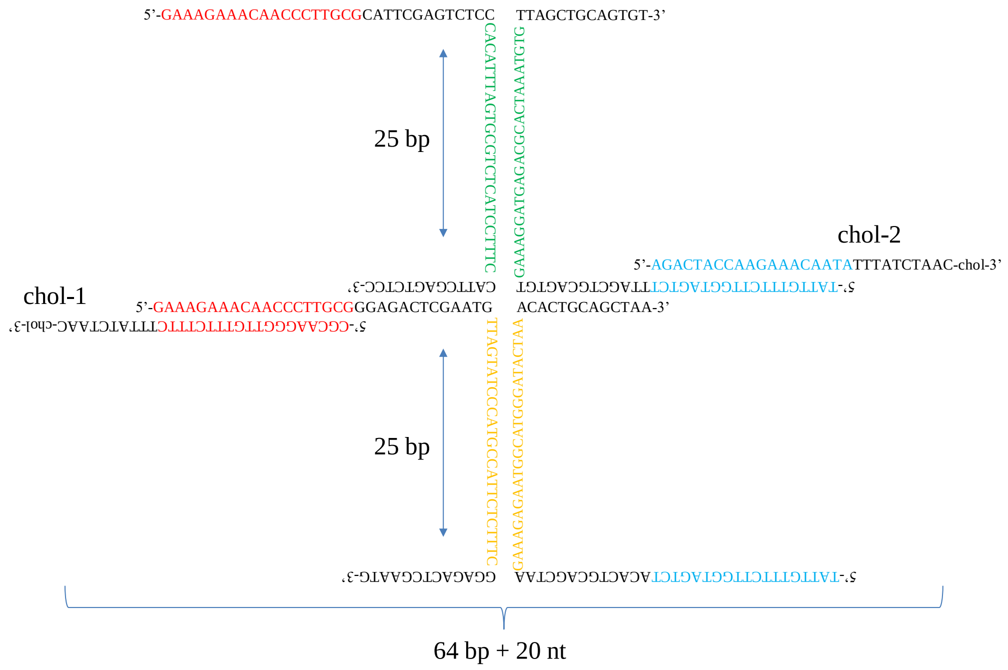

(E) Short side arm DNA nanobrush with backbone 21 bp ligated cholesterol (b4-2chol)

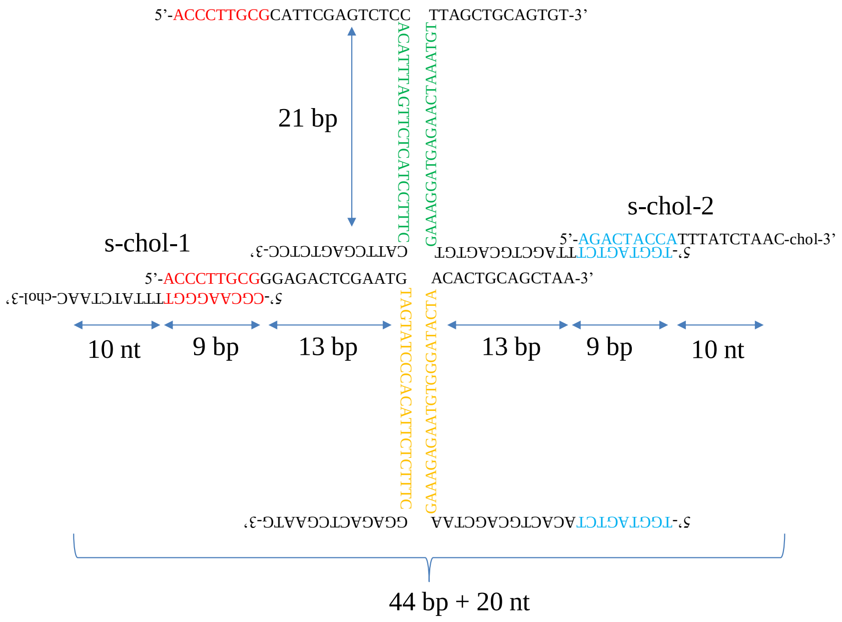

(F) DNA nanobrush with backbone 22 bp ligated one side cholesterol (b2-1chol)

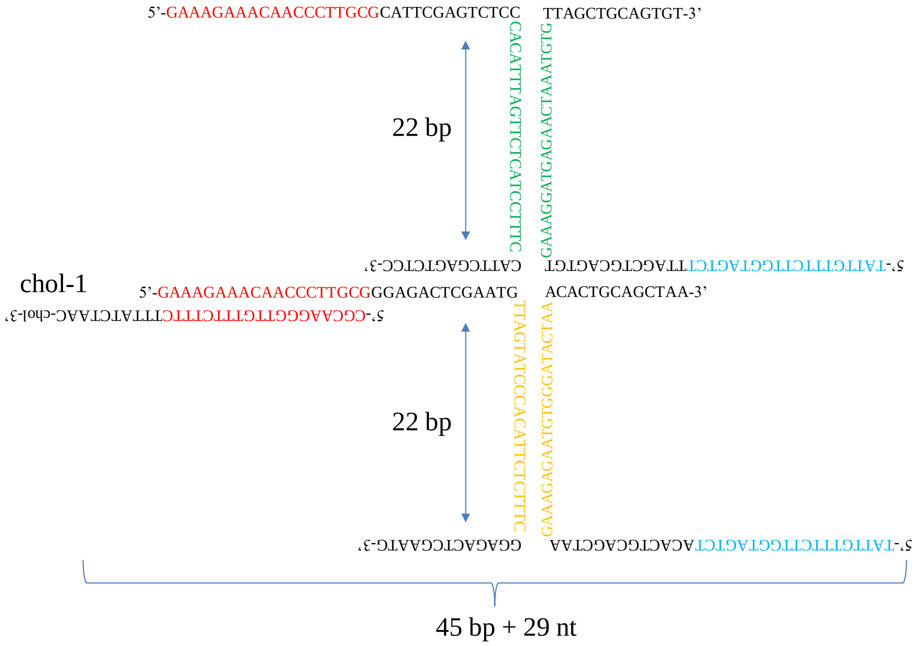

Figure S1. Schematic diagram of DNA nanobrush. (A) The nanobrush with a backbone 21 bp is a planar structure (b1). (B-D) Changing the backbone bases of the nanobrush to regulate the recognition direction. The nanobrush with a backbone 21 bp is a planar structure. The nanobrush with a backbone 25 bp is a completely twisted conformation. (E) Nanobrush with the short arm where the backbone is 21 bp (b4), both side arm connects cholesterol (b4-2chol). (F) Nanobrush with backbone 22 bp, one side arm connects cholesterol (b2-1chol).

Synthesis and characterization of the DNA nanobrush

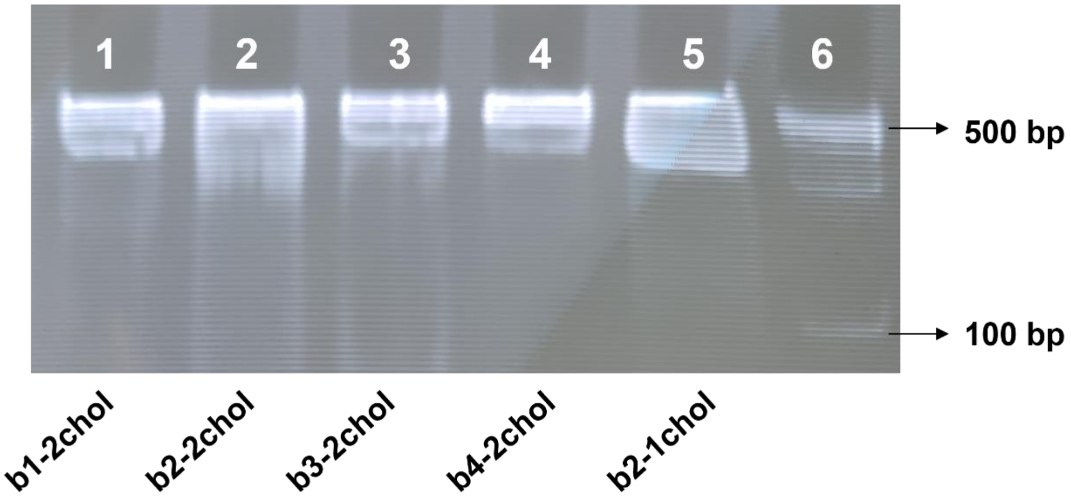

Figure S2. 10% polyacrylamide gel electrophoresis (PAGE) gel assay of different DNA nanobrushes. From lanes 1 to 6: b1-2chol; b2-2chol; b3-2chol; b4-2chol; b2-1chol; DNA ladder. Due to the increased size of DNA nanobrush formation, 10% PAGE gel cannot accurately distinguish the subtle differences between different DNA nanobrushes, which still need to be characterized by MIET experiments in this paper.

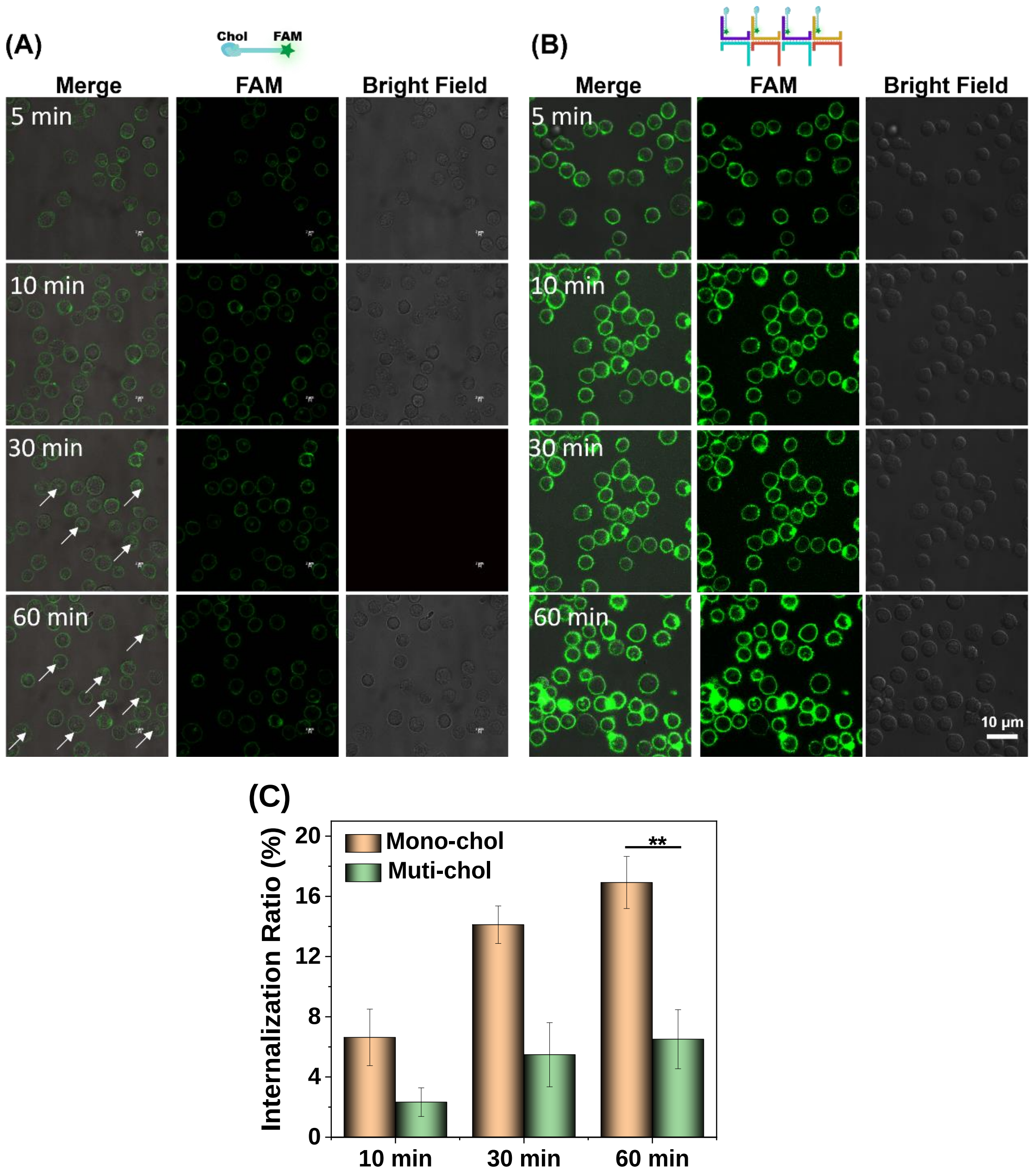

Figure S3. Internalization of nanobrush under long-time incubation. Confocal laser scanning microscopy (CLSM) images showed the internalization of monovalent (A) and multivalent (B) cholesterol. Reactions were performed at 37 °C for different times in a medium containing 10% FBS. (C) Relative cell internalization rate analysis was performed on the corresponding samples. **P ≤ 0.01 by unpaired two-tailed t-test. For the statistical analysis, over 100 cells were measured with Image J software for each sample group, and at least three independent experiments were performed.

Optimization of two kinds of cells assembled by nanobrush

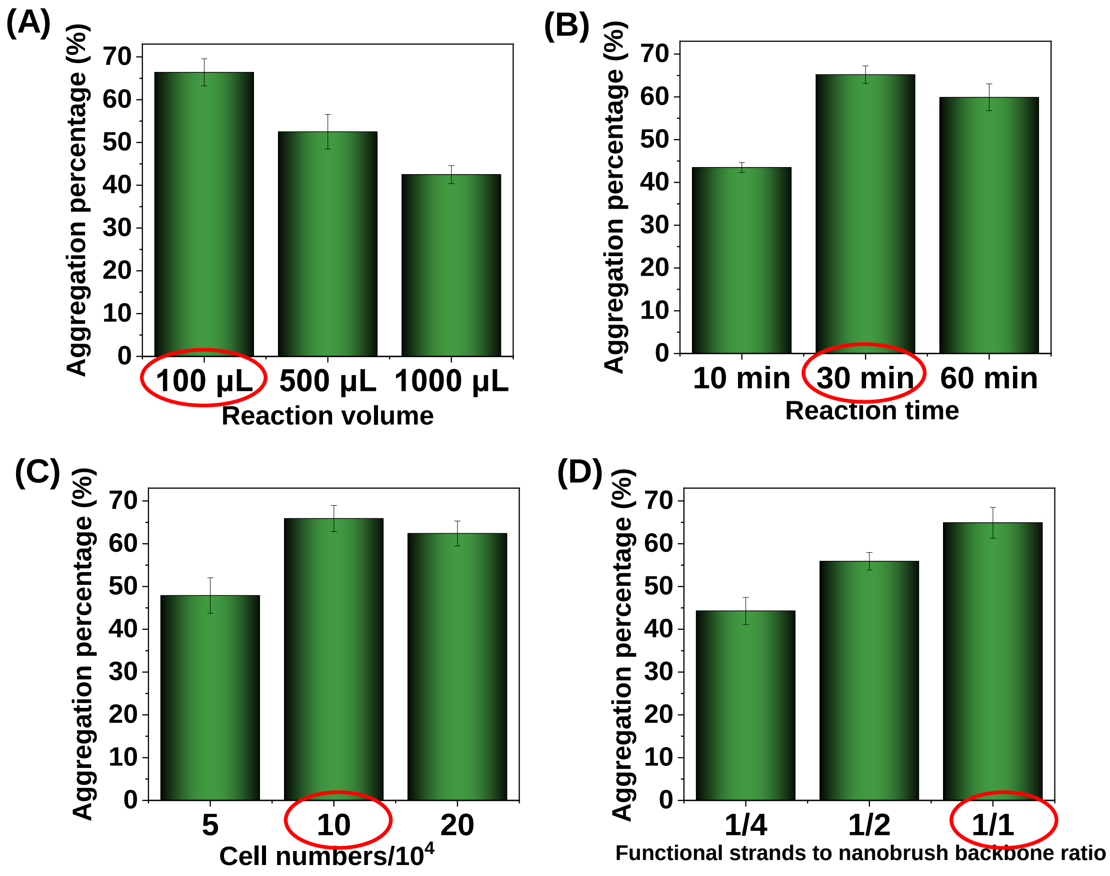

Figure S4. Optimizing the reaction conditions during two kinds of cell assembly: reaction volume (A); reaction time (B); cell number (C); the proportion of functional strands to nanobrush backbone sites. The optimal reaction conditions are (marked with red circle): 1×10^5 cells were reacted in 100 μL for 30 min. Also, the assembly efficiency is maximized when the functional strand completely occupies each side arm of the backbone extension. Bars represent mean ± SD (*n* = 3).

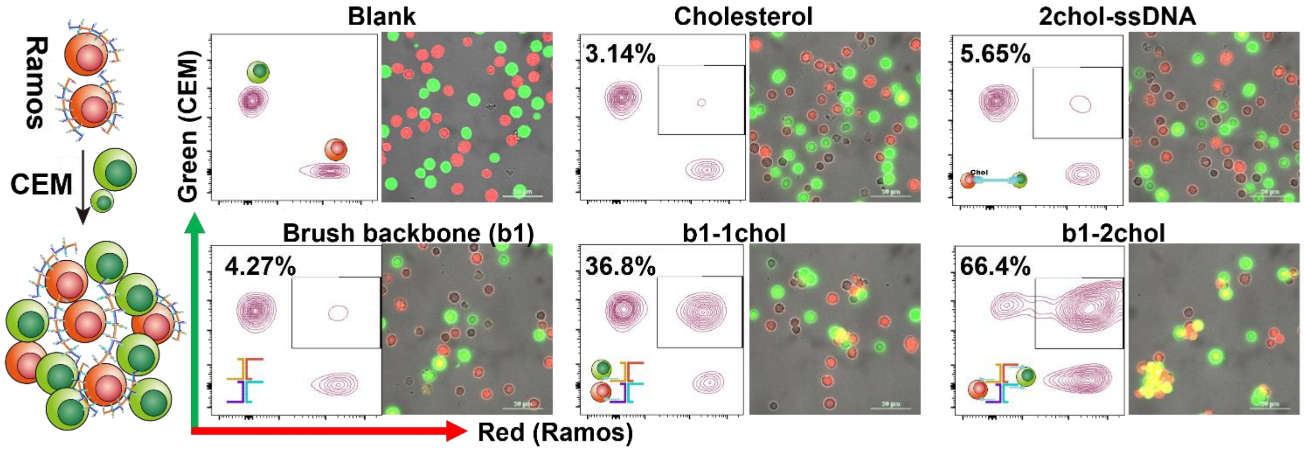

Figure S5. Aggregation of CCRF-CEM and Ramos cells assembled with different DNA nanobrushes. Characterization was performed using confocal microscopy and flow cytometry, respectively. Green: CCRF-CEM cells; Red: Ramos cells. As revealed by confocal microscopy imaging and flow cytometry, the two cells did not assemble without the addition of nanobrush. Similar results also appeared in the samples directly added cholesterol (commercial reagents) or added nanobrush backbone. When ssDNA labeled cholesterol at both ends (2chol-ssDNA) was added, the assembly efficiency was only 5.65%, which might be attributed to the inability of the single strands to form aggregates and fail to bring the two cells closer together. when using single-side arm linked cholesterol (b1-1chol) or double-sided arm linked cholesterol (b1-2chol), the assembly efficiencies can reach 36.8% and 66.4%, respectively. We can adjust the concentration of DNA nanobrush, reaction time and the number of functional groups, thereby controlling the size and proportion of cell assembly.

Stability of assembly of CCRF-CEM cells and nanobrush under long-term incubation

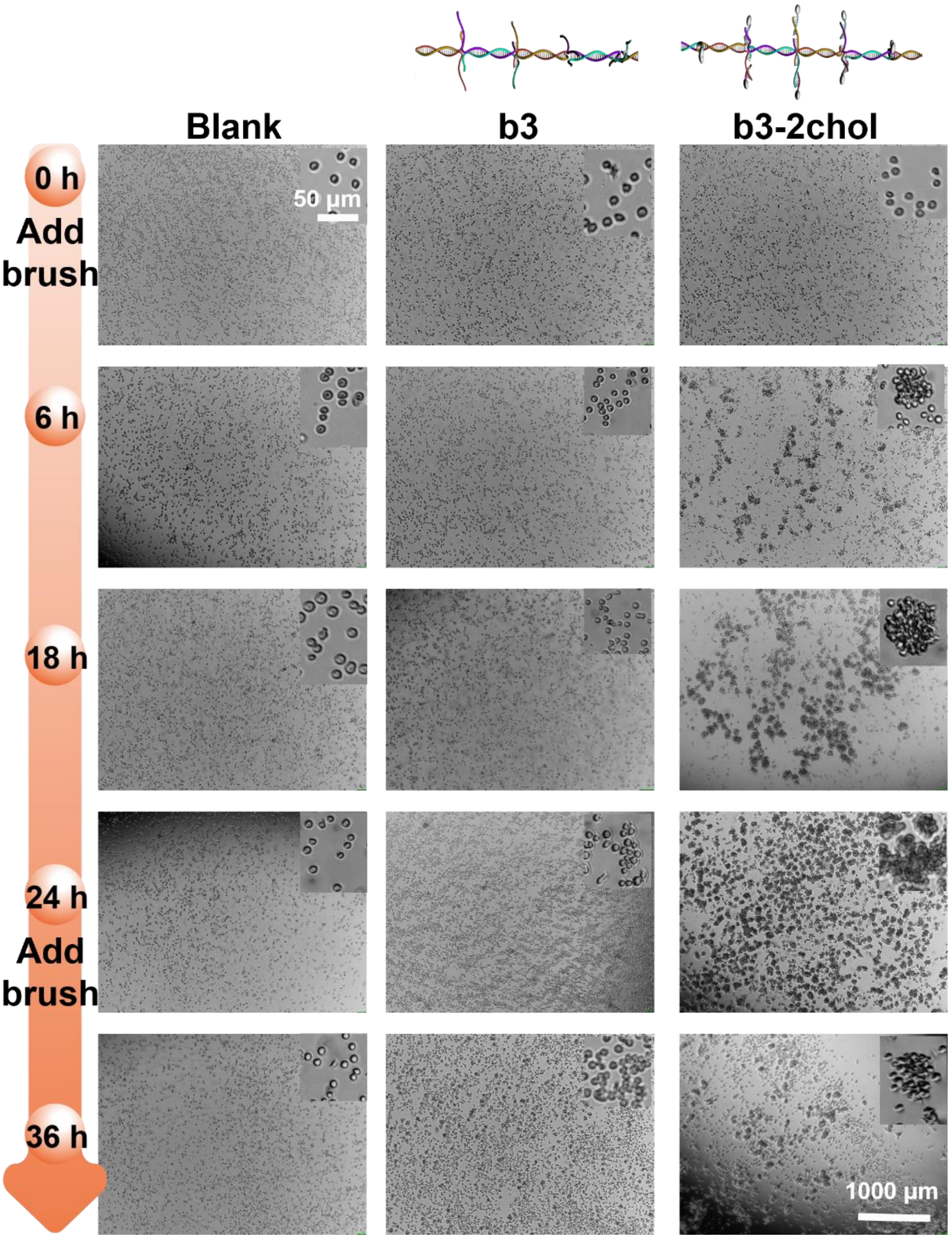

Figure S6. Stability experiments of DNA nanostructure-assembled cell clusters at different times. Initially, the experiment was divided into three groups: a blank control; DNA nanostructure backbone (b3, without functional group, 500 nM); b3-2chol (500 nM). As time progressed, cells in the blank and b3 groups dispersed individually without forming any noticeable clustered state. In contrast, the b3-2chol group exhibited stable cell assembly, maintaining a clustered cellular morphology. Beyond 24 h, the addition of nanobrush (500 nM) was required to reintroduce stability to the clusters. To facilitate cell growth, RPMI 1640 medium (GIBCO) containing 10% fetal bovine serum (FBS) and 1% antibiotics (penicillin-streptomycin-amphotericin B) was utilized. The cells were incubated at 37 °C in a CO_2 incubator. This result shows that even in the presence of 10% FBS, the nanostructures can remain stable in clusters for up to 24 h.

CCRF-CEM cells assembled into clusters with different nanobrush

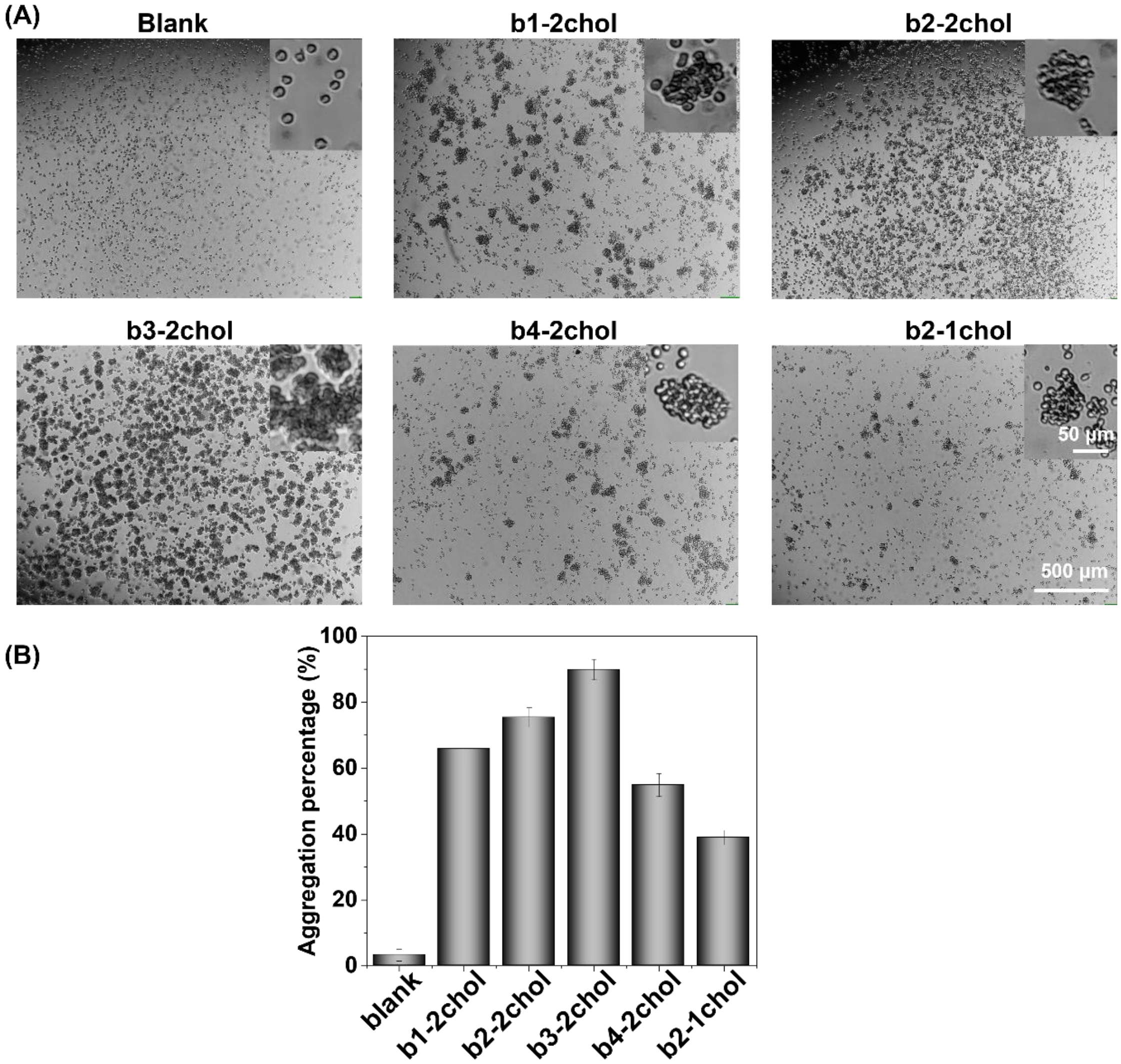

Figure S7. Aggregates of CCRF-CEM cells were assembled using various nanobrush structures and incubated for 24 hours. (A) The assembled cell groups were characterized using bright-field cell microscopy. Each sample contained the same number of cells. In the case of aggregated cell groups, all cells within the sample chamber tended to aggregate together. Consequently, when focusing on a fixed selection area, the cell density appeared higher due to this clustering effect. (B) Cell aggregation percentage for each nanostructure was quantified after 24 hours of incubation. It's worth mentioning that the assembly ratios (B) are slightly higher than those in Figure S4 due to the extended incubation period.

The results demonstrate the stable assembly of cell clusters by each nanobrush over an extended period. As the DNA backbone underwent gradual twisting, assembly efficiency also progressively increased. We speculate that this twisting exposed the contact sites of each functional chain, leading to more reactive sites, larger contact areas, and higher assembly ratios. The assembly efficiency of the nanobrush with a shortened arm (b4-2chol) was observed to be lower than that of b1-2chol. This disparity is attributed to the reduced length of the side arm, resulting in greater steric hindrance and diminished assembly efficiency. Despite having half the side arm length, the nanobrush (b2-1chol) could still assemble a proportion of cells due to its rotated structure. However, the absence of half the functional chains significantly reduced the assembly efficiency. These findings suggest the capability of distinct nanobrush structures to regulate intercellular assembly spacing, efficiency, and orientation. While the bright-field images provide insights into the nuanced variations in assembly ratios due to different nanobrushes, more comprehensive characterization is essential and should be validated through MIET results.

Characterize the smoothness and uniformity of MIET substrate surface

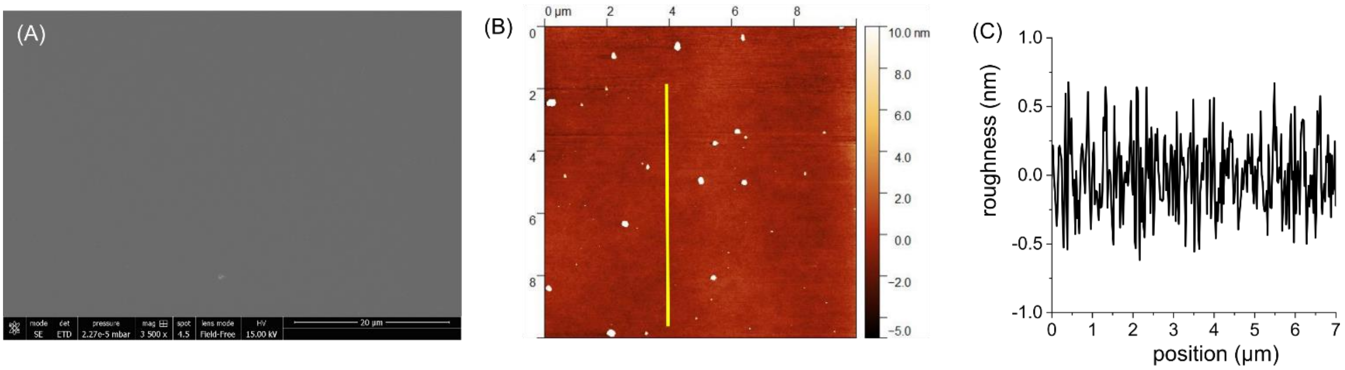

Figure S8. (A) Scanning electron microscopy (SEM) image. (B) Atomic force image (AFM) and (C) the corresponding surface roughness profiles of such MIET substrate. We obtained a root-mean-square value of roughness as 0.8 nm. These surface characterizations affirm that the MIET substrate exhibits exceptional smoothness and uniformity.

Scheme of MIET experimental set-up

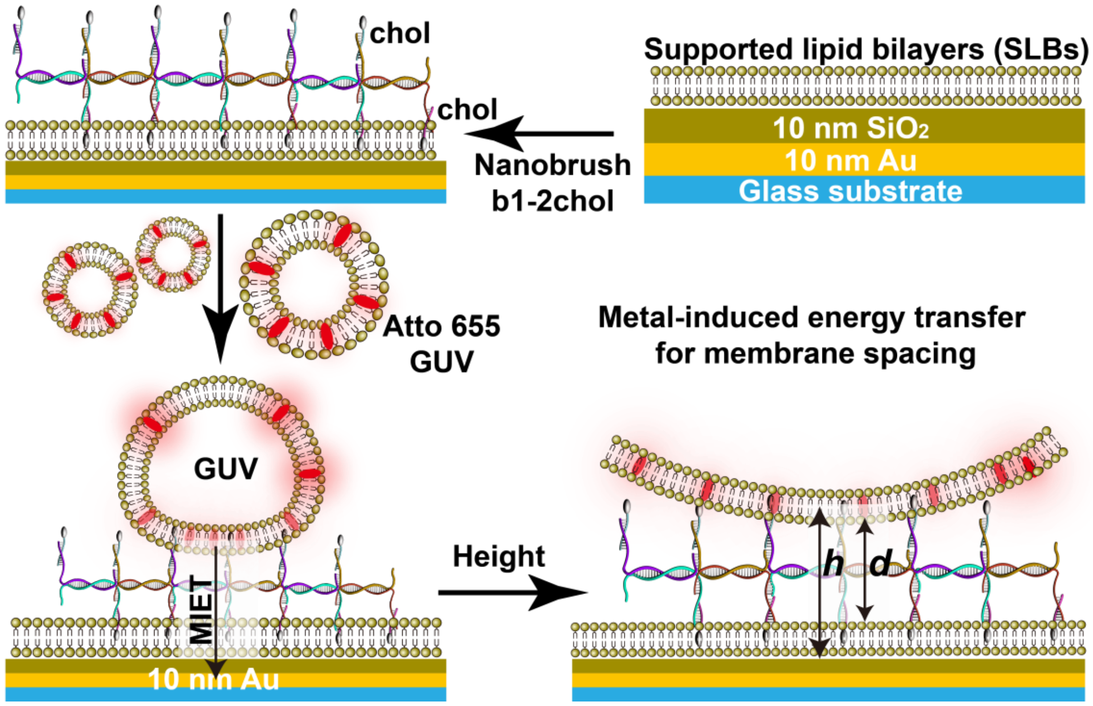

Figure S9. Scheme of the experimental set-up. The SLBs and GUVs were used to detect the distance between the two membranes assembled by the nanobrush.

MIET measurements of DNA nanobrush-assembled membranes

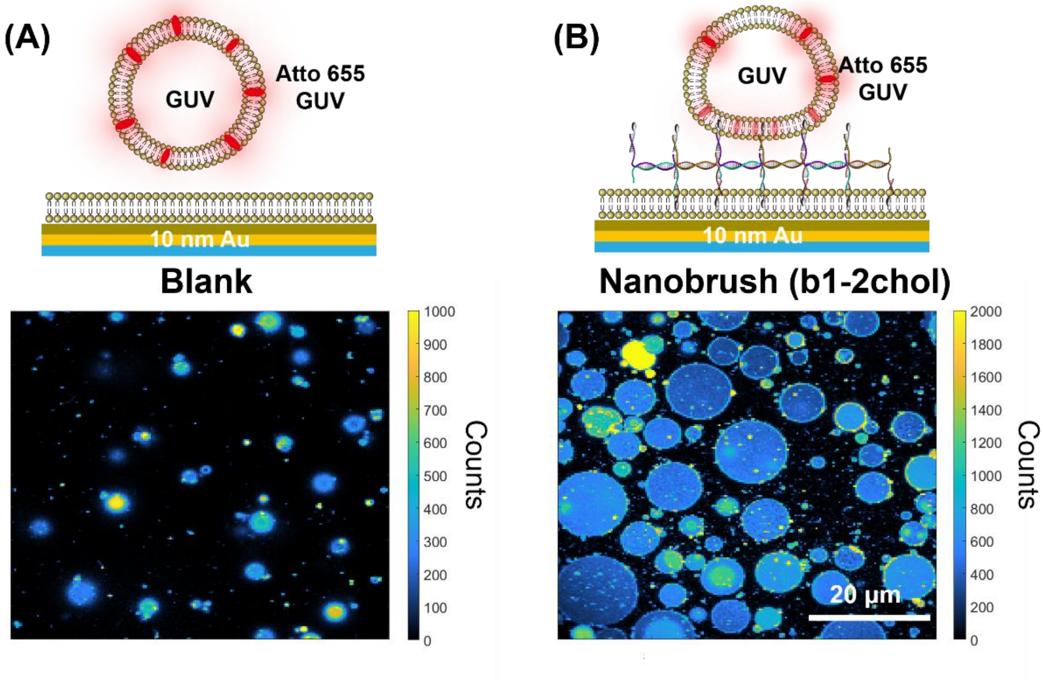

Figure S10. Fluorescence intensity images showed the adherence of the GUVs membrane without (A) or with (B) the addition of nanobrush. At least three independent experiments were performed.

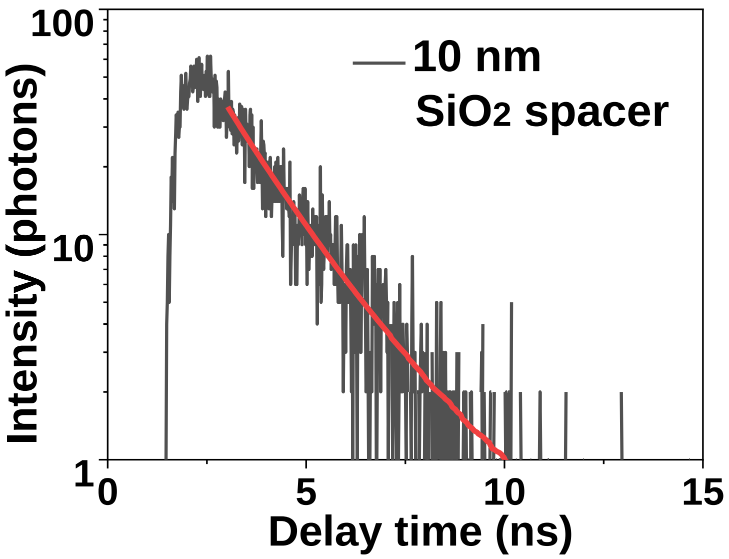

Figure S11. Typical time-correlated single photon counting (TCSPC) histogram of a single pixel (black). Multi-exponentially fit (red) to obtain the fluorescence lifetime.

Cryo-TEM to visualize the inter-membrane distance regulated by DNA nanobrush

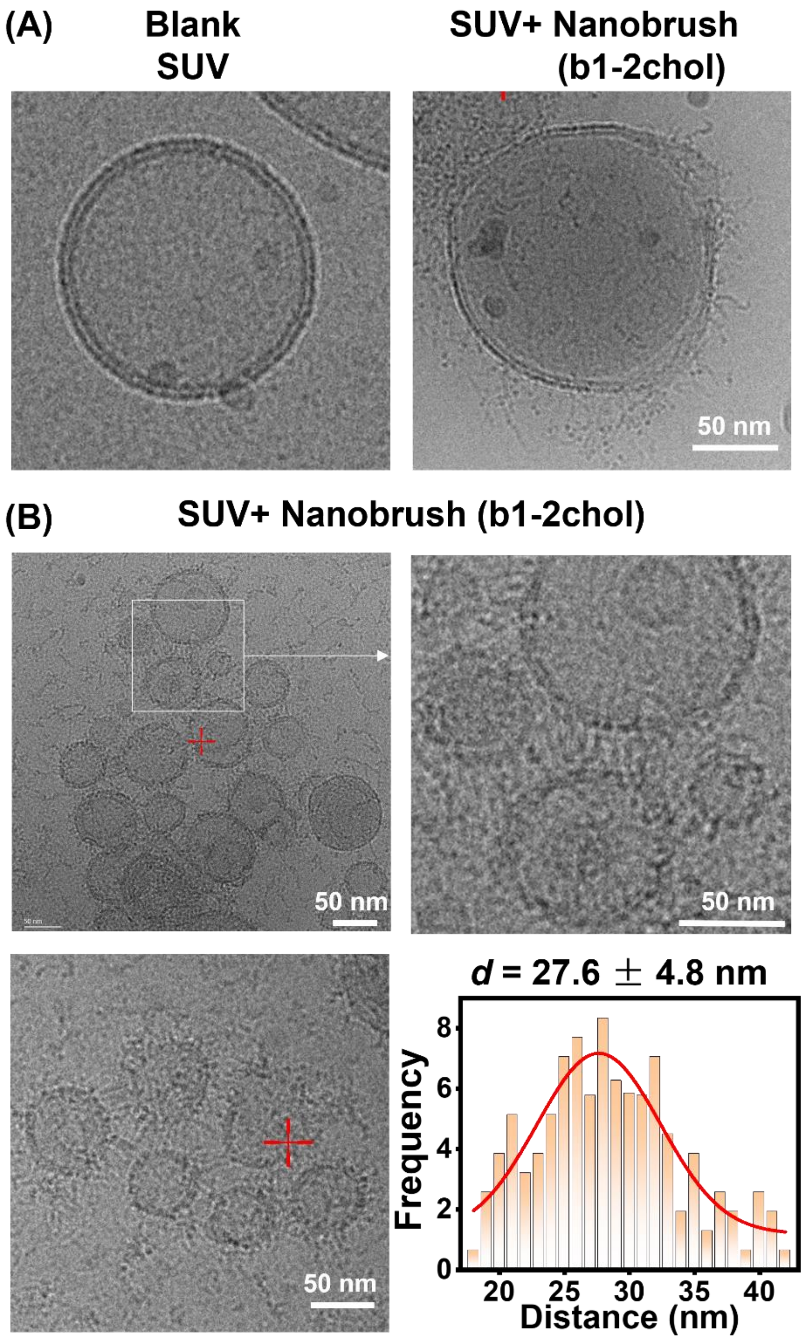

Figure S12. Cryo-TEM to visualize the inter-membrane distance regulated by DNA nanobrush. (A) Small unilamellar vesicles (SUVs) are depicted without (left) and with (right) the DNA nanobrush (b1-2chol). (B) Quantitative measurement of SUVs distance after adding nanobrush (b1-2chol). Membrane spacing was quantified using Image J, with red curves indicating Gaussian fits of the distance distributions, and the fitted distance is 27.6 nm ± 4.8 nm.

MIET microscopy to visualize the binding process using NIH-3T3 cell

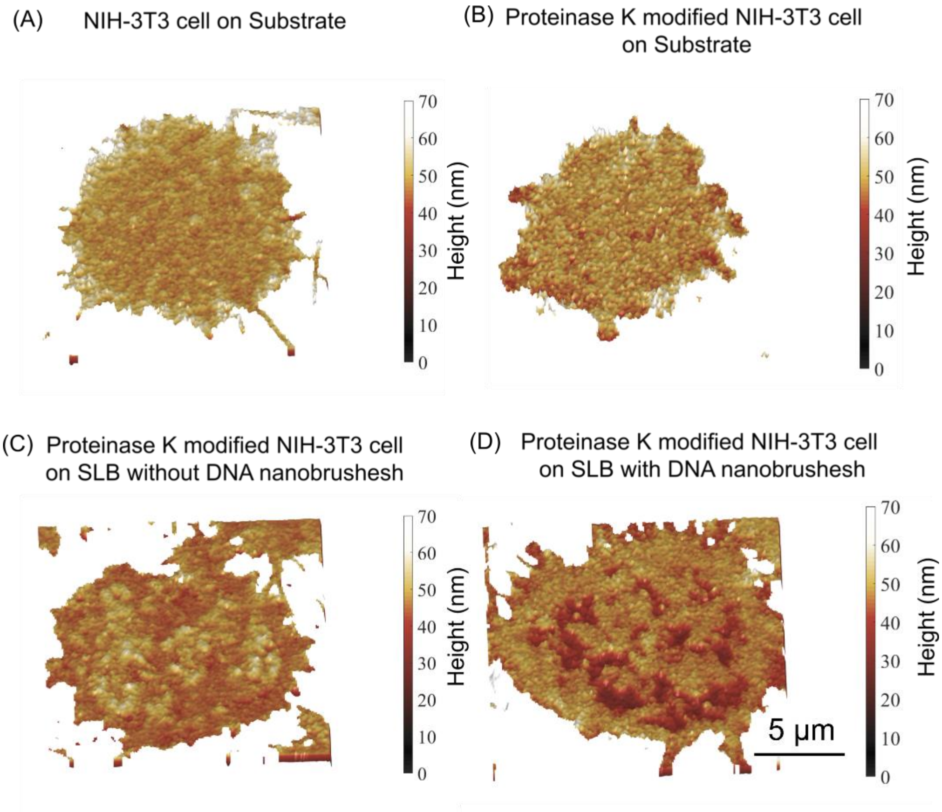

Figure S13. (A) Reconstruction of the 3D height (*h*) maps of the basal membrane of a NIH-3T3 cell on MIET substrate without SLB or DNA nanostructures. (B) Reconstruction of the 3D height (*h*) maps of the basal membrane of a proteinase-K-treated NIH-3T3 cell on MIET substrate without SLB or DNA nanostructures. The proteinase K enzyme used is to remove the surface proteins of the plasma membrane. (C) Reconstruction of the 3D height (*h*) maps of the basal membrane of a proteinase-K-treated NIH-3T3 cell on SLB supported on MIET substrate without DNA nanostructures. (D) Reconstruction of the 3D height (*h*) maps of the basal membrane of a proteinase-K-treated NIH-3T3 cell on SLB supported on MIET substrate with DNA nanostructures. The images were captured following a 60-minute incubation period of the cell on the substrate or SLB. The images suggest that although the removal of surface proteins from the plasma membrane decreased the membrane-membrane distance (panel A and panel B), adhesion between the plasma membrane and the supported lipid bilayer (SLB) didn't occur without DNA nanobrushes (panel C). Conversely, adhesion was observed in the presence of DNA nanobrushes (panel D).

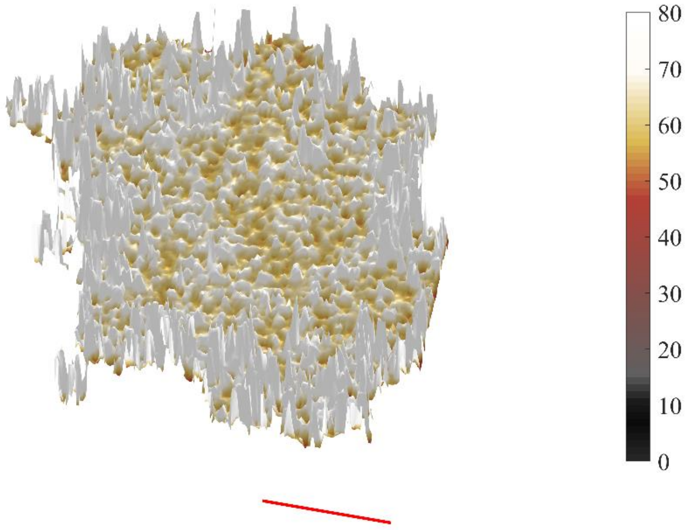

Figure S14. Three-dimensional height reconstruction of NIH-3T3 cells at 60 min in the absence of nanobrush. The scale bar is 5 μm.

MIET microscopy to visualize the binding process using COS-7 cells

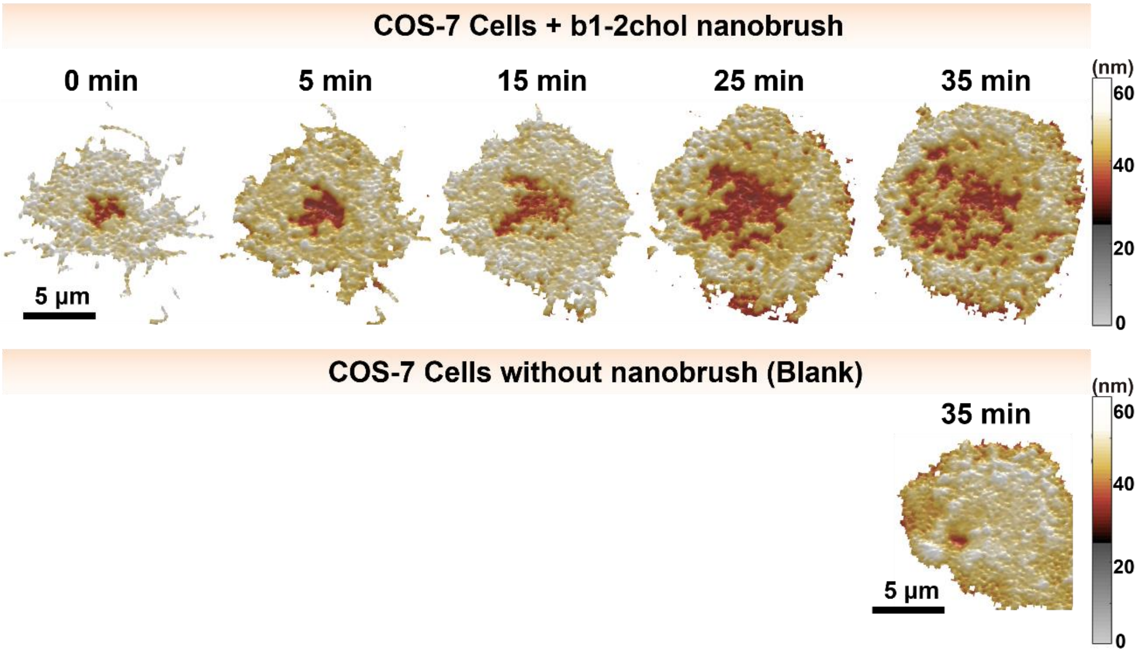

Figure S15. Measurement of DNA-nanobrush regulated binding of a COS-7 cell to an SLB with MIET imaging/spectroscopy. Reconstruction of the 3D height (*h*) maps of the proximal membrane of a COS-7 cell mediated with or without DNA nanostructures. For each image, the photons are accumulated for 40 s.

MIET microscopy to visualize the binding process using U_2Os cells

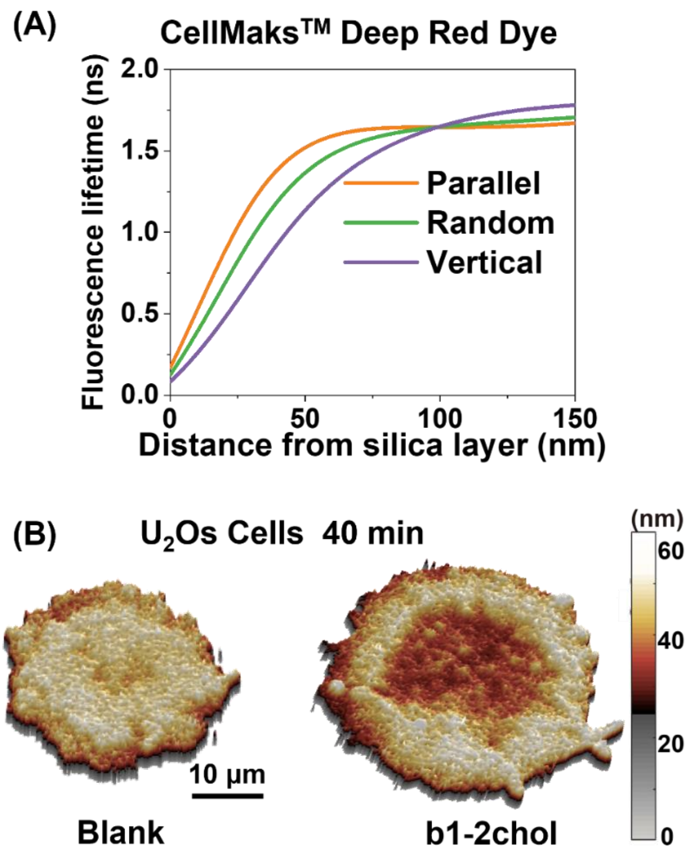

Figure S16. Measurement of DNA-nanobrush regulated binding of a U_2Os cell to an SLB with MIET imaging/spectroscopy. To further expand the application of MIET in the detection of cell membrane surface engineering, we use commercial cell membrane dyes (CellMaks^{TM} Deep Red) to demonstrate the general applicability of the method. (A) Calculated dependence of fluorophore lifetime on its axial position over the silica surface (commercial dye: CellMaks^{TM} Deep Red plasma membrane stain, free-space lifetime = 1.75, quantum yield = 0.30). Curves are calculated for an emission wavelength of 680 nm. 10 nm gold and 10 nm SiO_2 deposited on glass cover slide. (B) Reconstruction of the 3D height (h) maps of the proximal membrane of a U_2Os cell mediated by DNA nanostructures. The scale bar is 10 μm.

References

[1] Chizhik, A. I.; Rother, J.; Gregor, I.; Janshoff, A.; Enderlein, J. Metal-Induced Energy Transfer for Live Cell Nanoscopy. *Nat. Photonics* **2014**, *8*, 124-127.

[2] Baronsky, T.; Ruhlandt, D.; Bruckner, B. R.; Schafer, J.; Karedla, N.; Isbaner, S.; Hahnel, D.; Gregor, I.; Enderlein, J.; Janshoff, A.; Chizhik, A. I. Cell-Substrate Dynamics of the Epithelial-to-Mesenchymal Transition. *Nano Lett.* **2017**, *17*, 3320-3326.

[3] Chizhik, A. M.; Ruhlandt, D.; Pfaff, J.; Karedla, N.; Chizhik, A. I.; Gregor, I.; Kehlenbach, R. H.; Enderlein, J. Three-Dimensional Reconstruction of Nuclear Envelope Architecture Using Dual-Color Metal-Induced Energy Transfer Imaging. *ACS Nano* **2017**, *11*, 11839-11846.

[4] Chizhik, A. M.; Wollnik, C.; Ruhlandt, D.; Karedla, N.; Chizhik, A. I.; Hauke, L.; Hahnel, D.; Gregor, I.; Enderlein, J.; Rehfeldt, F. Dual-Color Metal-Induced and Forster Resonance Energy Transfer for Cell Nanoscopy. *Mol. Biol. Cell* **2018**, *29*, 846-851.

[5] Raja, S. O.; Chizhik, A. I.; Schmidt, C. F.; Enderlein, J.; Ghosh, A. Mapping Activity-Dependent Quasi-Stationary States of Mitochondrial Membranes with Graphene-Induced Energy Transfer Imaging. *Nano Lett.* **2021**, *21*, 8244-8249.

[6] Ghosh, A.; Chizhik, A. I.; Karedla, N.; Enderlein, J. Graphene- and Metal-Induced Energy Transfer for Single-Molecule Imaging and Live-Cell Nanoscopy with (Sub)-Nanometer Axial Resolution. *Nat. Protoc.* **2021**, *16*, 3695-3715.
